# Lineage-Specific Conserved Noncoding Sequences of Plant Genomes: Their Possible Role in Nucleosome Positioning

**DOI:** 10.1093/gbe/evu188

**Published:** 2014-09-05

**Authors:** Nilmini Hettiarachchi, Kirill Kryukov, Kenta Sumiyama, Naruya Saitou

**Affiliations:** ^1^Department of Genetics, School of Life Science, Graduate University for Advanced Studies (SOKENDAI), Mishima, Japan; ^2^Division of Population Genetics, National Institute of Genetics, Mishima, Japan; ^3^Department of Biological Sciences, Graduate School of Science, University of Tokyo, Japan

**Keywords:** conserved noncoding sequence, CNS, eudicots, monocots, angiosperms, plants

## Abstract

Many studies on conserved noncoding sequences (CNSs) have found that CNSs are enriched significantly in regulatory sequence elements. We conducted whole-genome analysis on plant CNSs to identify lineage-specific CNSs in eudicots, monocots, angiosperms, and vascular plants based on the premise that lineage-specific CNSs define lineage-specific characters and functions in groups of organisms. We identified 27 eudicot, 204 monocot, 6,536 grass, 19 angiosperm, and 2 vascular plant lineage-specific CNSs (lengths range from 16 to 1,517 bp) that presumably originated in their respective common ancestors. A stronger constraint on the CNSs located in the untranslated regions was observed. The CNSs were often flanked by genes involved in transcription regulation. A drop of A+T content near the border of CNSs was observed and CNS regions showed a higher nucleosome occupancy probability. These CNSs are candidate regulatory elements, which are expected to define lineage-specific features of various plant groups.

## Introduction

Since the emergence of studies based on conservation in the noncoding regions, numerous findings have been made on conserved noncoding sequences (CNSs) in animals (Bejerano et al. 2004; Sandelin et al. 2004; Vavouri et al. 2007; Lee et al. 2010), plants (Kaplinsky et al. 2002; Guo and Moose 2003; Inada et al. 2003; Kritsas et al. 2012), and even bacteria (Marchais et al. 2009). Many studies have concentrated the attention on animal CNSs which revealed that very high conservation is seen in the noncoding regions, they are under purifying selection (Casillas et al. 2007), and also they are found close to genes that act as developmental regulators (Sandelin et al. 2004; Woolfe et al. 2005; Vavouri et al. 2007; Elgar 2009; McEwen et al. 2009; Lee et al. 2010; Takahashi and Saitou 2012; Matsunami and Saitou 2012; Babarinde and Saitou 2013). A recent study (Lee et al. 2010) on ancient vertebrate CNSs in bony vertebrate lineages stated that the CNSs found are in abundance in tissue-specific enhancers and they are likely to be *cis*-regulatory elements that are functionally conserved through evolution. Furthermore, *Caenorhabditis elegans* has a unique set of CNSs that are not found in vertebrates and are associated with nematode regulatory genes (Vavouri et al. 2007). Drake et al. (2006) reported that the CNSs are not mutational cold spots but actually under selective constraint. These findings are based on the premise that conservation in the noncoding regions is coupled with regulation of genes, thus implying that the conservation could necessarily be due to a functional constraint.

These studies have set various selection criteria for the CNSs, such as the length and *e* value in BLAST homology searches. Irrespective of the selection criteria, in general, animal CNSs tend to be much longer and more frequent than plant CNSs, and plant CNSs are shorter than animal CNSs. Kaplinsky et al. (2002) reported that out of the seven orthologous genes compared on for rice and maize, six contained CNSs and all the CNSs were less than 60 bp long. Guo and Moose (2003) surveyed orthologous gene pairs in rice, maize, sorghum, and barley and considered 20 bp as the minimal criterion for a CNS in grasses. Inada et al. (2003) conducted a similar study for 52 orthologous genes of rice and maize considering 1,000 bp or more toward the promoter region of each gene, and found that most CNSs were short (<20 bp) although some were longer than 80 bp.

On the contrary to the previous studies that were restricted to a limited set of orthologous regions, Kritsas et al. (2012) analyzed complete genomes of four species (*Arabidopsis thaliana*, *Vitis vinifera*, *Brachypodium distachyon*, and *Oryza sativa*) and identified ultraconserved-like elements (ULE). They identified 36 highly conserved elements with at least 85% identity and are longer than 55 bp between *Ar. thaliana* and *V. vinifera* and also 4,572 highly conserved elements between *O. sativa* and *Brac. distachyon*. D’Hont et al. (2012) compared *Brac. distachyon*, *O. sativa*, and *Sorghum bicolor* and identified 16,978 CNSs which were defined as pan-grass CNSs. Further with the addition of *Musa acuminata*, they identified 116 CNSs in the commelinid monocotyledon lineage. Both these studies have concentrated on CNSs that are commonly found in the groups of species in their analyses, and also these CNSs might also be present in other lineages. The main focus of our study was to identify lineage-specific CNSs in a broader scale with species belonging to different taxonomic groups with the premise that these persistently conserved regions retained across extremes of various plant lineages may comprise critical regulatory modules, specific to these lineages. In our analysis, we identified and examined the lineage-specific CNSs that solely originated in their common ancestors based on the species used in the study; therefore, our result gives a broader view and understanding of lineage specificity of the CNSs and also adds information and value to plant CNS analyses, being the comprehensive investigation on lineage specificity for plant CNSs.

Nevertheless, all the above-mentioned studies imply that conservation irrespective of long divergence times exists due to various biological implications that are yet to be fully explained. With the ever increasing amount of high throughput data on complete plant genomes, we decided to focus on whole-genome analysis of available plant genomes to identify lineage-specific CNSs. Our focus is in finding all the CNSs, thus find potential regulatory elements specific to different plant lineages.

We searched eudicot lineage-specific CNSs by analyzing genome sequences of the following seven eudicot species: *Ar. thaliana*, *Brassica rapa*, *Populus tricocarpa*, *Ricinus communis*, *V. vinifera*, *Cucumis sativus*, and *Aquilegia coerulea.* To determine grass-specific CNSs, genome sequences of *O. sativa*, *Brac. distachyon*, *So. bicolor*, and *Setaria italica* were compared. The genome sequences of *M. acuminata* were also analyzed to determine monocot-specific CNSs in addition to the four grass species mentioned above. It has to be noted that in order to look for the specific CNSs in the analysis we have included the most basal species sequenced so far, assuming that if a CNS is present in the most diverged species, it is highly likely to be found in closer species inside a group. The most basal eudicot species used in the study is *Aq. coerulea* which diverged about 120 Ma (Anderson et al. 2005) from the rest of the eudicot species used in this study. *Musa acuminata* is considered as the basal monocot species, which diverged from grasses about 115 Ma (D’Hont et al. 2012). The other species used in the study are *Selaginella moellendorffii* that diverged from angiosperms about 400 Ma (Banks et al. 2011), *Physcomitrella patens* that diverged 450 Ma (Rensing et al. 2007) from vascular plants, and *Chlamydomonas reinhardtii* that diverged from land plants more than 1,000 Ma (Heckman et al. 2001). A total of 15 species (see fig. 3 for their phylogenetic relationship) were used with the expectation of finding the group-specific CNSs in this study.

In this analysis, we identified 27 eudicot, 204 monocot, 6,536 grass, 19 angiosperm, and 2 vascular plant lineage-specific CNSs. Untranslated region (UTR) CNSs showed a stronger constraint and also we found that these CNSs were flanked by genes involved in transcription regulation. We also observed a drop of A+T content in the borders of the CNSs and high nucleosome occupancy probability for the CNS regions.

## Materials and Methods

### Genomes Considered in the Analysis

Repeat masked genome sequences of *Ar. thaliana*, *Bras. rapa*, *Po. tricocarpa*, *O. sativa*, *Brac. distachyon*, *So. bicolor*, *Sel. moellendorffii*, *Ch. reinhardtii*, and *Ph. patens* were downloaded from Ensembl release 12, whereas *R. communis*, *V. vinifera*, *Cu. sativus*, *Aq. coerulea*, and *Set. italica* were downloaded from Phytozome version 8.0. Genome sequences of *M. acuminata* were downloaded from banana genome project database. As the analysis was focused on the CNSs in the nuclear DNA, the mitochondria and chloroplast genomes were removed from the analysis where they were known and annotated in the databases. As there is also a possibility of mitochondrial and chloroplast sequences being transferred into nuclear genome, we further removed any sequences which showed homology to mitochondrial or the chloroplast genome.

### Identification of Common CNSs

#### Common to Eudicots

BLAST 2.2.24+ (Altschul et al. 1997) was used for performing homology searches in this study. BLASTn search was done with *Ar. thaliana* as the query and *Bras. rapa* as the subject database. The cut off *e* value for the search was 0.001. Only the alignments without any overlap with a coding region for both query and subject were used for subsequent analysis. From the remaining (nuclear DNA) BLAST hits, the best hits of overlapping alignments were selected using the *e* value. If the coordinates of two sets alignments overlapped with each other, only the alignment with the lower *e* value was retained. Thus, a data set with the best alignments for *Ar. thaliana* and *Bras. rapa* CNSs was produced for further analysis. The obtained best hits were searched against *Cu. sativus*, thus *Ar. thaliana*, *Bras. rapa* and *Cu. sativus* best hits were obtained the same method explained above. Similarly, the best hits of *Ar. thaliana*, *Bras. rapa*, and *Cu. sativus* were searched against *Po. tricocarpa*. This method was carried out in form of a chain (best hits of previous step used to search a new species) for the following species, *R. communis*, *V. vinifera* and *Aq. coerulea* in the sequence given, to obtain the common CNSs to eudicots. These CNSs were in turn searched in Rfam v10.1 (June 2011) and the CNSs with overlaps with noncoding RNA were removed from further analysis.

#### Common to Grasses

BLASTn search was done with *O. sativa* as the query and *Brac. distachyon* as the subject database. The cut off *e* value for the search was 0.001. Only the alignments without any overlap with a coding region for both query and subject were used for subsequent analysis. The remaining hits were filtered based on the *e* value and only the best hits were retained to search against *Set. italica*. Then, *O. sativa*, *Brac. distachyon*, and *Set. italica* best hits were searched against the last monocot genome, *So. bicolor*. This procedure finally achieves the CNSs that are found in all the monocots used in the study and thus were considered as grass common CNSs. The common CNSs were searched in Rfam v10.1 (June 2011) and the CNSs with overlaps with noncoding RNA were removed from further analysis.

#### Common to All Monocots

The grass-common CNSs discovered from the previous step were searched in *M. acuminata* to obtain CNSs that are common in all monocot species used in the study. The cut off *e* value for the search was 0.001.

#### Common to All Angiosperms*,* to All Vascular Plants*,* and to All Plants

The eudicot common and monocot common CNSs were searched against each other with a cut off *e* value of 0.001, using the eudicot common CNSs as the query and the monocot common CNSs as the subject, and the best hits selected based on the *e* value were searched in *Sel. moellendorffii* which is a lower vascular plant in order to identify the CNSs common to vascular plants. The best hits from this step were searched in *Ph. patens* to identify any CNSs that could still be remaining as common to all land plants. Finally, the best hits from this step were searched in *Ch. reinhardtii* with the expectation to find any noncoding sequences conserved in the group Viridiplantae irrespective of their long divergence time.

### Identification of Lineage-Specific CNSs

#### Eudicot*,* Monocot*,* Angiosperm*,* and Vascular Plant Lineage-Specific CNSs

**Fig. 1.—:**
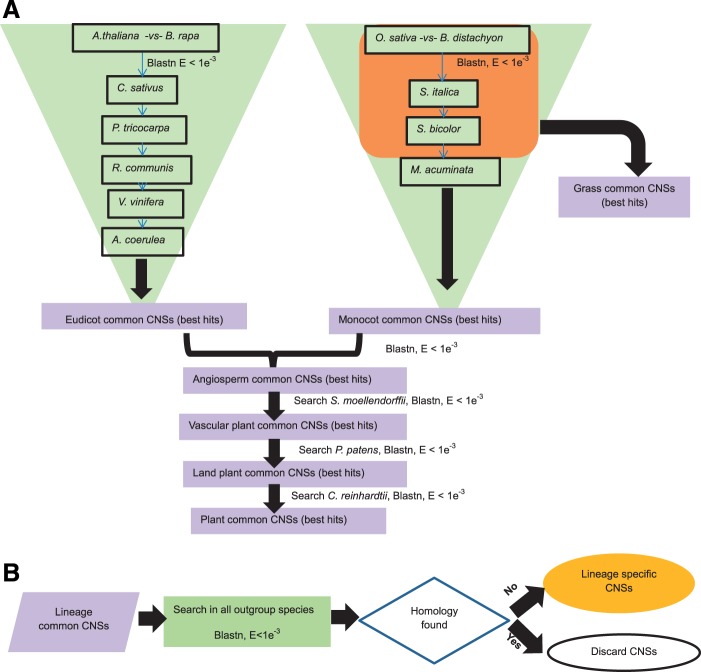
The flowcharts of the lineage-specific CNS determination. (*A*) Flowchart for lineage common CNS determination. (*B*) The flowchart for lineage-specific CNS determination.

All the eudicot common CNSs found were searched in all the outgroups used in the study (all the monocot species, *Sel. moellendorffii*, *Ph. patens*, and *Ch. reinhardtii*). The CNSs that are common to eudicots and not found in any of the outgroups were designated as eudicot-specific CNSs. Similarly, in order to identify monocot-specific CNSs, the monocot common CNSs were searched in the following outgroups, all the eudicots, *Sel. moellendorffii*, *Ph. patens*, and *Ch. reinhardtii* used in the study. The angiosperm-specific, vascular plant-specific, and plant-specific CNSs were identified the same way by searching against their outgroups. The flowchart for the analysis is depicted in figure 1.

#### Lineage-Specific Loss of CNSs

One main reason for the differences in abundance of lineage-specific CNSs is partially due to retention or loss of ancestral CNSs. Consideration of ancestral CNSs gives a comprehensive outline of the dynamics of the retention or loss of CNSs. To study the loss of ancestral CNSs, we considered *Ch. reinhardtii* as the basal species for all land plants for this analysis. We conducted independent homology searches for all the ingroup species, namely *Ar. thaliana*, *Bras. rapa*, *R. communis*, *Po. tricocarpa*, *Cu. sativus*, *V. vinifera*, *Aq. coerulea*, *Set. italica*, *So. bicolor*, *Brac. distachyon*, *O. sativa japonica*, *M. acuminata*, *Sel. moellendorffii*, and *Ph. patens* with *Ch. reinhardtii* as the query genome. Hits overlapping with any genes were filtered out and then a superset of all the ancestral CNSs found in all ingroup species was made by merging overlapping hits. This superset represents the aggregate of ancestral plant CNSs that are still found in *Ch. reinhardtii.* Based on this set of CNSs, the ancestral CNSs lost in each branch were found.

### Identification of CNSs for All Pairs of Species

**Fig. 2.—:**
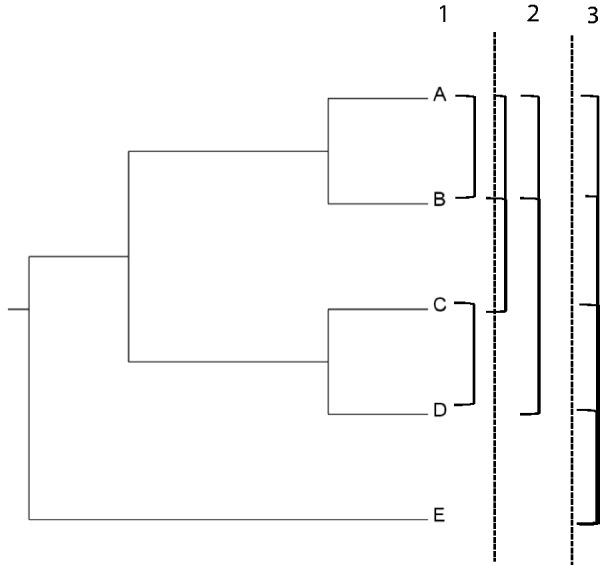
Example schematic diagram for identification of CNSs for all pairs of species. This schematic example with five species (A–E) shows how the pairwise searches are performed to determine the union of CNSs for each pair of species and separate lineages. In the example, the searches are performed in three levels (1–3). The bars on the right side connecting species represent separate searches. For all the species used in the analysis, a total of 105 searches were performed in a similar manner to determine the CNSs present in all pairs.

We conducted an analysis to determine CNSs that are present in all pairs of species and their common ancestors to provide a comprehensive view on presence of CNSs in each pair of species. In total, 105 searches were performed for this analysis between different pairs of species. The cut off *e* value for the search was 0.001. Only the alignments without any overlap with a coding region for both query and subject were considered. The schematic diagram for this analysis is depicted in figure 2.

### Analysis of Protein-Coding Genes

#### Predicted Target Genes of CNSs

The gene that lies closest to a particular CNS was considered as the likely target gene. If a CNS was found inside a gene in intron or UTR, the gene it resides was considered as the likely target gene. The likely target gene is with respect to the reference genomes used in the study. For monocot- and grass-specific CNSs, *O. sativa japonica* was considered as the reference genome and for eudicot-specific CNSs *Ar. thaliana* was the reference genome. These genomes have better genome annotation and quality, therefore were considered as the reference.

#### Identification of Lineage-Specific Genes or Orphan Genes

To establish a preliminary understanding of any correlation between lineage-specific CNSs and lineage-specific genes, we determined the numbers of lineage-specific genes for eudicots, monocots, and grasses. We considered protein-coding gene sequences of all eudicot and monocot species used in the analysis to run BLASTp searches. The cut off *e* value used was 0.00001 following Yang et al. (2013). The BLASTp searches were performed as in CNS search in a stepwise manner. The lineage-specific genes are defined as genes found in all the ingroup species but absent in all the outgroup species. It has to be noted that this analysis solely depends on the annotated protein-coding sequences and that we might have missed some unannotated genes.

#### Gene Enrichment Analysis for the Likely Target Genes

In order to identify the functional groups for the likely target genes, the gene enrichment analysis was carried out for grass- and monocot-specific CNSs using the Database for Annotation, Visualization and Integrated Discovery (DAVID) by Huang et al. (2009a, 2009b).

### Characterization of the CNSs

#### A+T Content in the Flanking Regions and the Inside of CNSs

Another analysis to characterize CNSs was carried out by exploring the A+T content in 1,000-bp flanking regions and the center (20 bp) of grass- and monocot-specific CNSs by a moving window analysis (10-bp window with 1-base step size). CNSs with flanking regions that ran into coding regions were removed from the analysis, altogether 4,993 grass-specific CNSs and 188 monocot-specific CNSs were considered for this analysis. The statistical significance was assessed by *t*-test.

#### Nucleosome Occupancy Probability

Kaplan et al. (2009) built a probabilistic model of sequence preferences of nucleosome regions. This model considers the dinucleotide signals along with specific pentamer sequences that are favored or disfavored in known nucleosome sequences to produce a score for each sequence under study. We downloaded their program from http://genie.weizmann.ac.il/software/nucleo_prediction.html (last accessed July 2014).

Nucleosome occupancy probabilities for grass- and monocot-specific CNSs were computed by considering a 4,000-base region to each side starting from the center of the CNSs by using this probabilistic model. The average nucleosome occupancy probability was then computed for both sides of each nucleotide site (in total of 8,000 sites) along the length of sequences. The same analysis was carried out for a random sample with same AT content as the CNSs (random sequences to have the same length as the CNSs) and also for a random sample with no specific AT preference. These random samples contained the same number of sequences as the CNSs and also same lengths with additional extending flanking regions. All the sequences were extracted from the noncoding regions of the rice genome. The average occupancy probability was calculated for all the 8,000 sites for all random sequences. Statistical significance was determined by using *t*-test.

#### CNSs and Recombination Hot Spots

The eudicot-specific CNSs were searched against recombination hot spot data for *Ar. thaliana* published by Horton et al. (2012).

#### Methylation Marks on Eudicot-Specific CNSs

Further methylation marks for eudicot-specific CNSs were determined by using bisulfite sequencing data for *Ar. thaliana* published by Cokus et al. (2008) and is available through http://epigenomics.mcdb.ucla.edu/BS-Seq/ (last accessed March 2014). Twenty-seven random samples of 27 sequences in each with the same lengths as eudicot-specific CNSs were extracted from the noncoding regions of *Ar. thaliana* and were searched for methylation marks. The eudicot-specific CNSs and the random samples were compared with two proportion *z* test at 95% confidence level.

#### Phylogenetic Tree Reconstruction with CNSs

The multiple sequence alignments were constructed for the lineage-specific CNSs. The aligned multiple sequences were concatenated and the neighbor-joining trees (Saitou and Nei 1987) for eudicots, grasses, monocots, and angiosperms were constructed with MEGA version 5 (Tamura et al. 2011).

Scripts used in this study and the sequence alignments can be made available upon request.

## Results

### Lineage-Specific CNSs

**Fig. 3.—:**
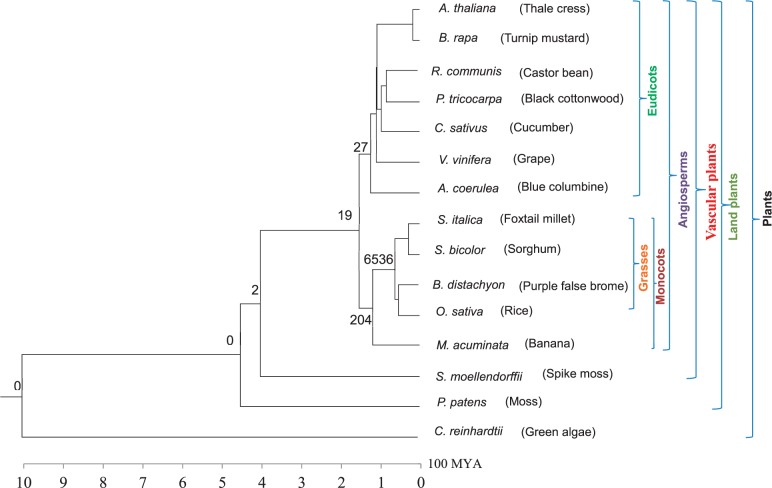
Phylogenetic tree with the number of lineage-specific CNSs. The numbers on each branch represent the number of lineage-specific CNSs found in the study. The main plant groups considered in the study are depicted on the right. The phylogenetic tree was constructed with verified divergence times taken from Anderson et al. (2005), D’Hont et al. (2012), Banks et al. (2011), Heckman et al. (2001), and Rensing et al. (2007).

**Fig. 4.—:**
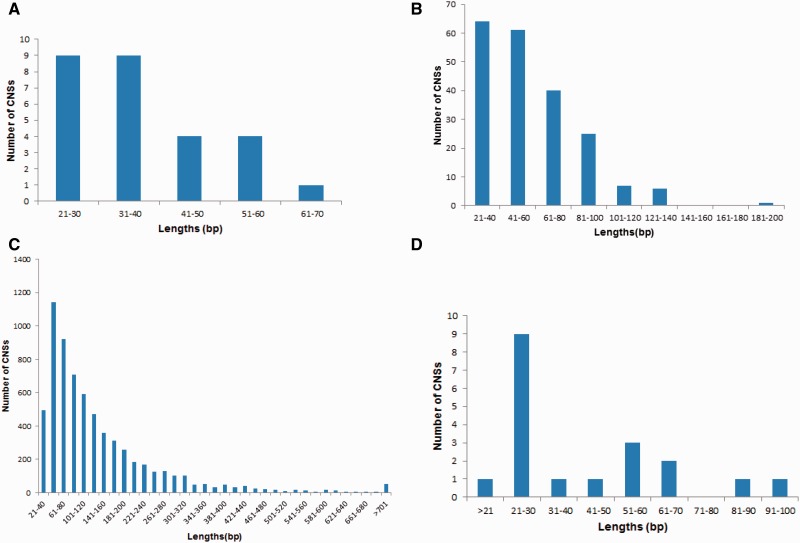
Length distributions of lineage-specific CNSs. (*A*) Length distribution for eudicot-specific CNSs. (*B*) Length distribution for monocot-specific CNSs. (*C*) Length distribution for grass-specific CNSs. (*D*) Length distribution for angiosperm-specific CNSs.

We identified 27 eudicot, 6,536 grass, 204 monocot, 19 angiosperm, and 2 vascular plant-specific CNSs (fig. 3). These lineage-specific CNSs are likely to have originated in their respective common ancestors. A large number of grass-specific CNSs were observed and as a whole monocots showed more lineage-specific CNSs than eudicots. The average lengths of lineage-specific CNSs are in the range of 35–60 bp except for grass-specific CNSs whose average CNS length was 140 bp (table 1). Length distributions of four lineage-specific CNSs are shown in figure 4. Although most of lineage-specific CNSs are shorter than 100 bp, 3,306 grass-specific CNSs and 14 monocot-specific were longer than 100 bp, and the longest grass-specific CNS was 1,517 bp (table 1). The minimum length for CNSs spans from 16 to 46 bp for all lineage-specific CNSs. The average percentage identity for all the lineage-specific CNSs was found to be more than 80% sequence similarity. CNS coordinates and sequences are provided in supplementary material files, Supplementary Material online, in FASTA format, and also multiple alignment results in supplementary material files, Supplementary Material online.

**Table 1:** Summary of Lineage-Specific CNSs: Minimum–Maximum Lengths, Average Lengths, Average Percentage Identity, and Number of CNSs Longer Than 100 bp

	Eudicot	Monocot	Grass	Angiosperm	Vascular Plant
	Specific	Specific	Specific	Specific	Specific
Number of CNSs	27	204	6,536	19	2
Minimum length (bp)	22	23	23	16	46
Maximum length (bp)	63	186	1,517	95	50
Average length (bp)	38.5	58.5	140.7	42.8	48.0
Average pid (%)	89.8	84.3	80.25	87.5	82.0
CNSs ≥ 100 bp	0	14	3,306	0	0

The average percentage identity for each length groupings for CNSs provided in supplementary figure S1, Supplementary Material online, shows that the shorter CNSs have higher percentage identity leading up to >90% and have a higher conservation level whereas the longer CNSs tend to have lower conservation level. It should be noted that distinction between one long CNS with varying degrees of conservation and a cluster of short CNSs separated with short nonconserved regions is not easy. Therefore, if we change the definition of CNSs by varying thresholds, the CNS length distribution may change.

The difference in the number of CNSs may be due to evolutionary rate differences of the lineages. In order to get an understanding whether evolutionary rate could contribute to the differences, we calculated the synonymous substitutions (Ds) between the reference genome and the most basal species inside the lineage. Eudicots have a very high saturated Ds value of 2.4363, whereas monocots and grasses have lower Ds values of 1.5118 and 0.6304, respectively. Therefore, evolutionary rate could possibly be one contributing factor for the heterogeneity in number of CNSs.

Can the short divergence time be the reason for the high number of grass-specific CNSs? To address this issue, we selected pairs of species from eudicots and grasses with approximately the same divergence time: *O. sativa* and *So. bicolor*—divergence time of 60–70 Ma (Woolfe et al. 1989), *C. papaya* and *Ar. thaliana*—divergence time about 70 Ma (Woodhouse et al. 2010). We then determined the number of CNSs for each pair. The eudicot pair had 1,324 CNSs, whereas the grass species pair had 16,029 CNSs. Even with the approximately similar divergence times, the pattern of CNSs remained the same as for the lineage-specific CNSs. We also determined whether the number of species used in the analysis for eudicots is responsible for the difference in the number of CNSs between eudicots and monocots. We randomly selected four eudicot species (*Bras. rapa*, *R. communis*, *Cu. sativus*, and *V. vinifera*) and determined the number of lineage-specific CNSs for them. The number of their lineage-specific CNSs was 118, which is still much less than the number of CNSs we obtained for grasses. We further selected five eudicot species (*Bras. rapa*, *Po. tricocarpa*, *R. communis*, *Cu. sativus*, and *V. vinifera*) which has a total of 120 Myr diveregence times, and determined the number of CNSs for them, in comparison with the number of CNSs obtained for the five monocot species in the study. The number of lineage-specific CNSs for those five eudicot species was 69, whereas the number of lineage-specific CNSs for five monocot species was 204. It should be mentioned that the tonal divergence time of these five monocot species is 115 Myr. Even with the same number and similar divergence times of eudicot and monocots, the number of eudicot-specific CNSs remains much less than monocot-specific CNSs. Therefore, the difference in number of CNSs is not due to the number of compared species.

**Fig. 5.—:**
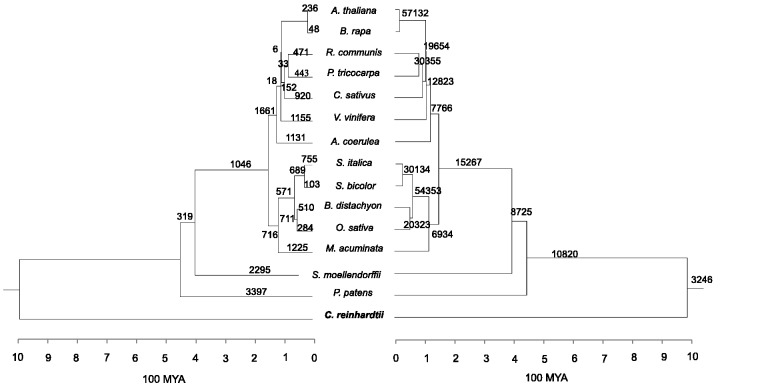
The lineage-specific loss of ancestral CNSs and the number of CNSs found from all pairwise searches. Left panel: The lineage-specific loss of ancestral CNSs. The values on branches represent the number of CNSs lost on that specific branch. The reference genome (*Ch. reinhardtii*) used for this analysis is highlighted in green. Right panel: Number of CNSs found from all pairwise searches. CNSs between all pairs of species were determined to have an overall comprehensive view on gain of noncoding conservation. These pairwise analyses consider the union of all CNSs. The number on each node reflects the gain of CNSs obtained through pairwise searches. These CNSs are common to each group of species and therefore are likely to be found in outgroup species.

It is important to note that our analysis started with an initial pair of species and try to determine lineage-specific CNSs for each group that are present only in all members of that lineage. But if we consider lineage common CNSs (CNSs that are commonly found in all members of a group but also might be found in outgroup species [supplementary fig. S7, Supplementary Material online]), it is clear that the lineage-specific CNSs are much less and represents a small fraction of elements that is likely to be functional in common ancestor. And further with our analysis to determine CNSs that are present in all pairs of species and their common ancestors (fig. 5 right panel, all searches conducted are given in supplementary table S5, Supplementary Material online), we found that the CNSs in each branch are higher (in this analyses, we considered the union of CNSs) than lineage common or lineage-specific CNSs. But many of these may have gone through independent losses inside a lineage, therefore would not fall under our criteria for determination of lineage-specific CNSs.

### Lineage-Specific CNSs and Lineage-Specific Genes

If the evolutionary rate had been a major contributing factor for the differences in the numbers of CNSs, the lineage-specific genes should also follow the same pattern as CNSs (unless the lineage-specific genes are under higher selective constraint). To investigate this scenario, we determined the numbers of lineage-specific genes and identified 2,439, 444, and 113 eudicot, grass, and monocot lineage-specific genes consecutively. The number of eudicot lineage-specific genes is much higher than grass and monocot lineage-specific genes, this is quite the opposite scenario to what was observed for lineage-specific CNSs. This observation gives light that apart from evolutionary rate there should be some other factors that contribute to the differences in CNSs. If we consider individual lineages, dicots evolved more lineage-specific genes whereas monocots and grass common ancestors gave rise to more lineage-specific CNSs.

We also found that these lineage-specific genes are predominantly plant defense related.

### Lineage-Specific Loss of Ancestral CNSs

One factor for the differences in the number of CNSs could be the loss or retention of ancestral CNSs by either rapid divergence or complete deletion (Hiller et al. 2012). Assuming an unbiased parallel loss between the reference genome (*Ch. reinhardtii*) and the other plant species, this analysis would provide an overall pattern on the rate of loss of CNSs of all plant species used.

We found that *Ch. reinhardtii* has 4,355 CNSs conserved in one or more of the species used. Based on this result, the number of CNSs lost in each branch was calculated. The result shows that eudicot common ancestor has lost twice as much CNSs than the monocot or grass common ancestors which indirectly answers the heterogeneity of the lineage-specific CNSs (fig. 5 left panel, supplementary table S1, Supplementary Material online). Of all the eudicots, *V. vinifera* and *Aq. coerulea* have lost the most number of CNSs independently in their respective lineages. It was observed that within grass lineage CNS loss has been lowered in *O. sativa japonica* and *So. bicolor.*

### The Genomic Locations of Identified Lineage-Specific CNSs

We examined the genomic locations of CNSs to see whether they are in UTR, intron, or in intergenic regions. Table 2 shows the frequencies of these three locations of the grass- and monocot-specific CNSs identified with respect to the genome of *O. sativa japonica* as the reference*.* The grass- and monocot-specific CNSs located in the intergenic regions (53.7% and 55%, respectively) are significantly less (*P* value for grass and monocots are 2.6E-161 and 0.000236, respectively) than the expectation from the genomic coverage, 70% for the reference rice genome. In contrast, the number of grass-specific and monocot-specific CNSs located in the UTR (25% and 22%, respectively) is significantly higher than the expectation from the genomic coverage (6%).

**Table 2:** Genomic Locations of the Lineage-Specific CNSs

	Rice Noncoding Genome Composition	Grass Specific	Monocot Specific	Arabidopsis Noncoding Genome Composition	Eudicot Specific	Angiosperm Specific
Intergenic	70.0	53.7 (3,503)	54.9 (112)	56.8	63.0 (17)	47.4 (9)
Intron	24.2	21.0 (1,374)	22.1 (45)	35.0	7.4 (2)	31.6 (6)
UTR	5.8	25.3 (1,658)	23.0 (47)	8.2	29.6 (8)	21.0 (4)
3′-UTR	3.4	19.2 (1,259)	11.3 (23)	4.0	14.8 (4)	10.5 (2)
5′-UTR	2.4	6.1 (399)	11.7 (24)	4.2	14.8 (4)	10.5 (2)

Note.—Genomic locations of grass and monocot-specific CNSs with respect to the reference genome *Oryza sativa japonica* are provided as a percentage in third and fourth columns. Rough percentage estimations of the intergenic, intron and UTRs for the reference genome are provided under rice noncoding genome composition in the second column. Genomic locations of eudicot and angiosperm-specific CNSs with respect to the reference genome *Arabidopsis thaliana* are provided as a percentage in sixth and seventh columns. Rough percentage estimations of the intergenic, intron, and UTRs for the reference genome are provided under *Arabidopsis* genome composition in the fifth column. The exact number of CNSs in each region is given in parentheses.

Although the eudicot and angiosperm-specific CNSs are less in number, the CNSs located in the UTRs followed a similar pattern having a significantly higher representation than the genomic coverage for the UTRs in the reference genome (table 2; *Ar. thaliana* was used as the reference genome). The result implies a stronger constraint on the CNSs located in the UTRs for the lineage-specific CNSs observed.

### Distribution of the CNSs in Chromosomes

We next examined chromosomal distributions of lineage-specific CNSs. Grass and monocot-specific CNSs were found distributed among all the 12 chromosomes with respect to *O. sativa japonica*—the reference genome (see supplementary figs. S3 and S4, Supplementary Material online, for grass-specific and monocot-specific CNSs, respectively). However, the numbers of CNSs on each chromosome varied and further, intrachromosomal distributions of the CNSs were observed to be uneven as well. One example is chromosome 10 with CNSs concentrated in several areas in reference genome for grass-specific CNSs (encircled in black—three clear clusters). This indicates that rather than being distributed randomly, CNSs tend to exist in clusters. A similar pattern was observed for monocot-specific CNSs. Some example likely target genes related to CNSs in cluster 3 in supplementary figure S3, Supplementary Material online, are, FAD-dependent oxidoreductase domain containing protein, aldo/keto reductase family protein, transcription factor BTF3, double-stranded RNA binding motif containing protein, cytokinin dehydrogenase precursor, etc. Functions of some of these genes are documented to be important in regulation of genes. Even though it has been reported that much of plant BTF3 functions still remain obscure, previous researches suggest that BTF3 is associated with HR (hypersensitive-mediated) cell death and involved in biotic stress regulation in the nucleus (Huh et al. 2012), and also double-stranded RNA binding protein plays a vital role in viral defense and development by regulation of cellular signaling events and gene expression (Waterhouse et al. 2001).

### Predicted Target Genes of CNSs and Their Enrichment Analysis

We consider the genes closest to the CNSs as the likely target gene based on the premise that the regulatory elements reside close to the gene it regulates.

The gene enrichment analysis for the predicted likely target genes of the grass- and monocot-specific CNSs indicates that these genes are predominantly involved in the regulation of transcription and DNA binding. Table 3 shows the top 20 groups in which genes are enriched in grass-specific and monocot-specific CNSs. The *P* values of the result suggest that the groupings are highly statistically significant. However, the gene ontology (GO) groupings obtained for a random sample of 6,536 genes (*O. sativa japonica*) also showed groupings with statistically significant *P* values up to *P* = 1.7 × 10^−^^8^ (supplementary table S2, Supplementary Material online). If we normalize the *P* values acquired for the actual data set shown in table 3 by dividing this randomly obtained *P* value, still all genes are highly statistically significant. As for gene enrichment for monocot-specific CNSs, now only the top seven groups become statistically significant after the same normalization. One important feature shared between grass-specific CNSs and monocot-specific CNSs is that they are predominantly enriched with genes related to transcription regulation. GO groups involved in enzymatic activity and various catabolic processes were significantly under represented, meaning that CNSs are less associated with such genes (supplementary tables S3*A* and *B*, Supplementary Material online). It has to be noted that functional classification is not an absolute decision-making procedure regarding the likely target genes of CNSs but rather indeed an exploratory method to identify the possible likely functions of flanking genes.

**Table 3:** Gene Enrichment Analysis for the Lineage-Specific CNSs

Functional Group	Percentage of Genes in the Group	*P* value
Likely target genes of grass-specific CNSs		
Functions related to nucleus	61.9	0.0E-0
Regulation of transcription	70.5	0.0E-0
DNA-binding	51.5	9.6E-309
Transcription	46.1	4.5E-278
Transcription regulator activity	69.1	5.7E-272
Transcription factor activity	65.6	8.6E-269
Regulation of RNA metabolic processes	41.7	4.6E-171
Zinc-finger related	23.3	3.4E-106
Activator	14.6	1.3E-86
Sequence-specific DNA binding	21.9	2.0E-83
Zinc ion binding	36.6	2.8E-72
Metal ion binding	25.1	1.1E-59
Homeodomain related	12.7	2.2E-54
Response to organic substance	24.5	1.5E-51
Myb-type HTH DNA-binding domain	10.0	5.6E-46
Cellular response to hormone stimulus	13.9	1.4E-43
Hormone mediated signaling	13.9	1.4E-43
Myb, DNA binding	10.3	1.8E-43
Pathogenesis-related transcription factor and ERF, DNA binding	7.7	3.9E-39
Transition metal ion binding	39.3	1.2E-38
Likely target genes of monocot-specific CNSs		
Transcription factor activity	92.3	2.4E-51
Transcription regulator activity	92.3	5.8E-48
Regulation of transcription	90.8	1.3E-46
Functions related to nucleus	72.3	5.8E-45
DNA binding	93.8	1.2E-43
Sequence-specific DNA binding	35.4	1.8E-17
Zinc-finger related	26.2	3.0E-12
Homeodomain related	16.9	3.6E-8
Basic-leucine zipper transcription factor	10.8	1.3E-7
Metal binding	27.7	1.3E-7
Activator	13.8	2.1E-7
Transcription factor, GATA, plant	13.8	6.2E-5
Myb-like DNA binding region	9.2	5.5E-6
No apical meristem protein	9.2	2.0E-5
Heat shock factor type, DNA binding	6.2	5.1E-5
Homeobox conserved site	7.7	1.4E-4
Anther development	6.2	1.6E-4
Androecium development	6.2	8.2E-4
Stamen development	6.2	8.2E-4
Postembryonic development	18.5	8.7E-4

Our result agrees with several animal CNS studies reported so far, stating that CNSs are found near genes involved in regulation of transcription and development (Hardison 2000; Sandelin et al. 2004; Shin et al. 2005; Venkatesh et al. 2006; Janes et al 2010). Kritsas et al. (2012) stated that their GO analysis showed that genes associated with *Ar. thaliana* ULE were involved in development and are likely to be developmentally regulated. Some of the eudicot-specific CNSs in our analysis were found close to transcription factors such as Kanadi2 which regulates embryo development (Heyndrickx and Vandepoele 2012), transcription factor jumonji which regulates circadian clock (Lu et al. 2010), and *DELLA* protein RGL1 which is a negative regulator of plant hormone gibberellin (Tyler et al. 2004). Seven likely target genes of eudicot-specific CNSs were found to be enzyme encoding. The two vascular plant CNSs were found in the intron region of the genes, suppressor of auxin resistance 3, and pre-mRNA splicing factor 38A. Plants deficient in suppressor of auxin resistance show pleiotropic growth defects, such as shorter primary root and fewer lateral root. And also it has been found that flowering occurs earlier than the wild type, flowers are smaller and less fit during life cycle (Parry et al. 2006). Further Parry et al. (2006) reported that this protein plays an important role in hormonal regulation and development in plants. Whereas pre-mRNA splicing factors and regulators are found to be quite important in splice site selection (Reddy et al. 2012) to ensure accurate transcript formation, including instances such as alternative splicing (Mabon and Misteli 2005).

We examined overrepresented motifs of lineage-specific CNSs using MEME (Bailey et al. 2009). Results (see supplementary table S4, Supplementary Material online) suggest that some motifs are related to genes with enriched GO terms (Buske et al. 2009) related to DNA binding and transcription factor activity.

### Synteny of Target Genes

We found all target genes of dicot-specific CNSs to have orthologs in *Aq. coerulea* genome (the most basal dicot species used in the analysis) in a 5-kb range from the *Aq. coerulea* CNSs (subject genome in this context) implying that CNSs are conserved along with the syntenic positions even in species with long divergence times. Similarly for grass- and monocot-specific CNSs, 5,770/6,536 and 170/204 target genes, respectively, had orthologs located in same syntenic positions along with the CNSs. This implies that CNSs and their target genes have been conserved as one block during evolution. The conservation level of the orthologous target genes was found to be statistically significant (*t*-test) when compared with random samples of orthologous genes (*P* values 1.05E-11, 5.87E-05, and 0.00, respectively, for eudicot, monocot, and grass orthologous genes).

### A+T Content in the Flanking Regions of CNSs

**Fig. 6.—:**
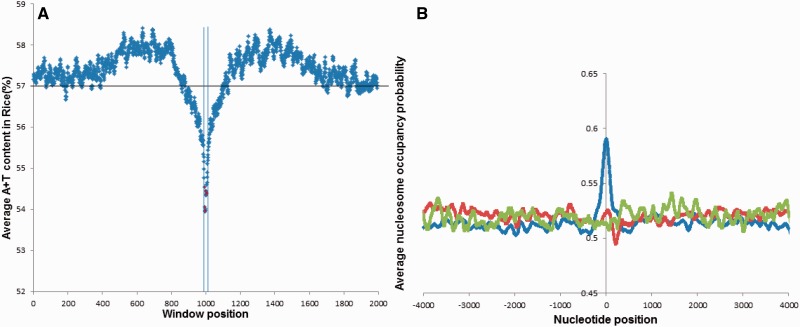
(*A*) Distribution of A+T content in the flanking regions and within CNSs (for grass-specific CNSs) black line—average A+T content in rice genome. Red dots—A+T content inside CNSs (20 bp from the center of each CNS was considered as mentioned in the methodology) acquired through moving window analysis. Blue vertical lines—borders of 5′ and 3′ flanking regions around the CNS. (*B*) Nucleosome occupancy probability for grass-specific CNSs including flanking regions. Zeroth nucleotide position represents the center of each CNS and also the center of the random samples. Blue, red, and green graphs, respectively, show nucleosome occupancy probabilities of the CNSs, random sample with same AT content as CNSs, and the random sample without specific AT preference.

To determine whether there are specific characteristics in CNSs and their flanking regions, the A+T content in the flanking regions and the inside of the CNSs were determined. In total, 1,000 bp of flanking regions in the 5′ and 3′ directions and 20 bp from the middle of the CNS were considered. A moving window analysis (10-bp window and 1-bp step size) was used to determine the A+T frequency around the margin and the inside of the CNSs for grass- and monocot-specific CNSs. A decline in the A+T content was observed near the start of the grass-specific CNSs as shown in figure 6*A*. A very similar pattern was observed for monocot-specific CNSs (supplementary fig. S5*A*, Supplementary Material online). The average A+T content of the flanking regions is 56%, which is same as the genomic A+T content of rice and the average A+T content of the CNSs is about 54%. *t*-Test showed that there is a statistically significant difference between the flanking regions and the CNSs with respect to the A+T content (*P* value <0.0005). Interestingly, the drop of A+T content at the flanking regions has also been observed in animal CNSs (Walter et al. 2005; Vavouri et al. 2007). Kritsas et al. (2012) also reported the similar scenario being present in plant CNSs, in which they considered *Ar. thaliana*, *V. vinifera* and *O. sativa*, *Brac. distachyon* to look for ULE in plants. This finding agrees with previous literature. It has to be noted that the feature of the drop of A+T frequency near the border of CNSs seems to be conserved between animals and plants similarly as was reported by Kritsas et al. (2012). A similar tendency of A+T drop was observed for eudicot-specific CNSs with a significant difference between the flanking regions and the inside of CNSs (95% confidence *P* value < 0.05). The lineage-specific CNSs we identified are GC rich compared with the genomic average. Babarinde and Saitou (2013) reported that animal CNSs in their analysis are GC poor, this finding stands opposing to our observation for plant CNSs.

### Prediction of Nucleosome Positioning

The A+T content can affect the DNA topology and nucleosome positioning. Jansen and Verstrepen (2011) showed that A+T rich sequences in *Saccharomyces cerevisiae* have a very low tendency to form nucleosomes. Nucleosome positioning pattern facilitates the access of transcription factors to their target sites and it plays a pivotal role in determining the transcription level (Bai and Morozov 2010). Therefore, the drop of A+T around the flanking regions of CNSs may be contributing toward nucleosome formation. We determined the nucleosome occupancy probability for the grass- and monocot-specific CNSs and their flanking regions with nucleosome prediction software by Kaplan et al. (2009). From the center of each CNS, a 4,000-bp region in 5′ and 3′ directions was considered. Two additional control samples were selected from the noncoding regions of the rice genome. A clear peak can be observed (fig. 6*B*) in the averaged nucleosome occupancy directly overlapping with the center and surrounding regions of the CNSs indicating a possibility of nucleosome positioning in the CNS regions. Even though a slight increase in the nucleosome occupancy probability can be seen in the random sample with the same AT content as the CNSs, the nucleosome occupancy probability of the CNSs is highly statistically significant compared with the random sample with same AT content (*P* = 2.80E-10). A very similar pattern was observed for the monocot-specific CNSs (supplementary fig. S5*B*, Supplementary Material online) again indicating a clear nucleosome positioning in and around CNSs. Similarly a high statistical significance (*P* = 0) was observed between the CNSs and the random sample with same AT content. The random samples for both grass- and monocot-specific CNSs with no AT preference stayed roughly constant with regards to the nucleosome occupancy probability throughout the length. One interesting feature observed for the UTR CNSs was the higher nucleosome occupancy obtained for 5′-UTRs in comparison with 3′-UTR CNSs. Nucleosome occupancy for 3′-UTR was almost similar to random expectation (supplementary fig. S8, Supplementary Material online). As reported by Bai and Morozov (2010), Jiang and Pugh (2009) nucleosome positioning is related to gene regulation and this result gives thorough evidence that CNSs can be involved in regulation of their target genes. Also Tillo et al. (2010) has reported that there is high nucleosome occupancy at regulatory sequences in the human genome. Also Baxter et al. (2012) observed a similar pattern of nucleosome positioning for CNSs in four eudicot plants.

### CNSs Are Not Related with Recombination Hotspots

It has been found that the A+T content might be related to recombination rates. Spencer et al. (2006) found that recombination hotspots for the human genome are associated with increased GC content or in other words lower A+T content. Guo et al. (2008) found that for rice and human genomes microsatellites with motifs consisting of only A and T such as AT, TA have lower recombination rates. As there was a decline in the A+T content in the flanking regions of the CNSs, we checked whether the eudicot-specific CNSs overlapped with any recombination hot spots that were found by Horton et al. (2012) for *Ar. thaliana* genome. We found that none of the CNSs overlapped with recombination hot spots documented in the above-mentioned study. An observation similar to ours was found for Kritsas et al. (2012) for eudicot ULEs. The decline in the A+T content may not be related to any recombination rate variation, but may be related to an unknown feature that is related to the function of the CNSs.

### Methylation in Eudicot-Specific CNSs

Methylation of the cytocine residues is a well-observed and understood phenomenon in many organisms including *Ar. thaliana*. DNA methylation is known to be related to regulation of gene expression and many other numerous cellular processes, such as embryonic development, genomic imprinting, and preservation of chromosome stability (Phillips 2008; Geiman and Muegge 2010; Gutierrez-Arcelus et al. 2013). To see whether the eudicot-specific CNSs contain any signature of methylation (Kritsas et al. 2012), those CNSs were compared with *Ar. thaliana* whole-genome methylation data obtained by Cokus et al. (2008). Only 2 of the 27 eudicot-specific CNSs showed methylation mark in CG, CHH, or CHG sequence contexts, in the minus or the plus strand. About 20% of the *Arabidopsis* genome is methylated including transposable elements and repeats (Bilichak et al. 2011). The methylation signature for the selected random samples provided sufficient evidence that the proportion of methylated CNSs is less than the proportion of methylated sequences in the random sample (*z* value for the test statistic is −2.66, *P* < 0.05). This result implies that the probability of observing methylation in eudicot-specific CNSs is less than the probability of observing methylation in the random samples at 95% confidence level according to two-proportion *z* test. Therefore, we conclude that eudicot-specific CNSs found in this study are not predominantly modified by DNA methylation.

### Phylogenetic Tree Construction with CNSs

We constructed the phylogenetic trees for each plant group considered in the study by using lineage-specific CNSs. The trees were constructed with 1,000 bootstrap replications and the model used was maximum composite likelihood method. The neighbor-joining trees (Saitou and Nei 1987) constructed for grass- and monocot-specific CNSs (supplementary fig. S6*A* and *B*, Supplementary Material online) exactly comply with monocot and grass phylogenies (Bennetzen et al. 2012) with greater than 80% bootstrap probability. Branching of *Set. italica* and *So. bicolor* showed 100% bootstrap value, whereas *O. sativa* and *Brac. distachyon* showed 80% bootstrap value for the tree constructed with monocot-specific CNSs. The expectation of phylogenetic tree construction with CNSs is that if the CNSs are orthologous it should be possible to reconstruct the species tree with high statistical support.

Even though we constructed similar trees for eudicot and angiosperm CNSs, only some branching showed high bootstrap values and agreed with the known phylogeny. *Arabidopsis thaliana* and *Bras. rapa* were always clustered with 100% bootstrap value in both eudicot and angiosperm trees (supplementary figs. S6*C* and *D*, Supplementary Material online). Monocots were also clustered together as expected with 100% bootstrap confidence.

The fact that the exact topology could not be achieved with eudicot and angiosperm CNSs does not mean that the CNSs are not orthologous, one reason for not being able to achieve the exact known topology could be that the concatenated sequence lengths for eudicot and angiosperm CNSs are too short (which were <1,500 bp) or in other words number of informative sites are less for phylogenetic tree construction. When sequences are short and the divergence among sequences is low, it affects the phylogenetic tree construction.

## Discussion

We identified lineage-specific CNSs that originated in their respective common ancestors in this study. These CNSs likely define their lineage-specific characters and functions. We observed a large number of CNSs that originated in the grass common ancestor and shared by all the grass species used in the study. One plausible reason for this high number could be the short divergence time of the grass species, but when compared with eudicot species with the same divergence time as the grasses the pattern of CNSs remained the same, in other words eudicots still had much less CNSs than grasses irrespective of the divergence time. This implies that grasses may have developed their own specific regulatory mechanisms to withstand diverse conditions such as biotic, abiotic stresses and to facilitate various other molecular processes. Furthermore, this suggests that grasses might be sharing similar kind of regulation as a group of species that might be contributing to their lineage-specific features.

In contrast, eudicots have much smaller number of lineage-specific CNSs shared by all the species used in the study. One explanation could be that many of the CNSs that originated in the eudicot-common ancestor have diverged beyond recognition, that they no longer can be detected by homology search. It is likely that the CNSs have degraded over evolutionary time but the binding functions may be conserved even though they cannot be detected by sequence alignment due to binding site turnover. And also whole-genome duplication events that occurred during evolution of eudicots can be another plausible reason for the less number of eudicot CNSs (Tang et al. 2008).

As the number of monocot-specific CNSs is 7-fold higher than eudicot-specific CNSs, it is possible that, after the divergence from common ancestor of angiosperm, monocots gained more CNSs to establish monocot-specific features, which are still found conserved in monocot species. This could be due to various physiological and morphological complexity differences between monocots and eudicots. The vascular bundle formation and arrangement is more complex in monocots than in eudicots, monocot embryogenesis differs broadly from that of eudicot and the architecture of monocotyledonous embryo is far more complex, and complexity in differences in root formation (multiple layers of cortical cells in monocot root whereas eudicots have one layer of cells; Grunewald et al. 2007) is some of the differences known with respect to complexity. Zimmermann and Werr (2007) reported that even at very early stages of monocot embryogenesis the cell division patterns are variable and unpredictable, further they report that primary root of cereals is formed endogenously deep inside the embryo which is a major difference with the dicots. And also the embryonic axis of the monocots is displaced laterally respect to scutellum in contrast to apical–basal axis of dicots (Zimmermann and Werr 2005). Also it has been reported that the shoot apical meristem differs in structure and function in eudicots and monocots (Sussex 1989; Jürgens 1992; Kerstetter and Hake 1997). Therefore, primarily, complexity could be one reason for monocots to have more CNSs as they require more regulation.

The fact that only two specific CNSs were found to be conserved in all the vascular plants suggests that in general, plant CNSs have a high turnover rate and many CNSs originated in vascular plant, land plant and plant common ancestors have diverged beyond recognition.

One interesting feature observed in our analysis is the difference in the numbers of lineage-specific CNSs and lineage-specific genes. The lineage-specific genes showed quite the opposite pattern to CNSs. The eudicots had the least number of CNSs but the highest number of lineage-specific genes, whereas grasses had the highest number of CNSs but lineage-specific genes were 5-fold less than that of eudicots. It appears that eudicots gave rise to more lineage-specific genes whereas grasses and monocots evolved more CNSs in their respective common ancestors. It would be very interesting to find out what factors actually govern organisms in originating genes and CNSs in their respective common ancestors. In other words, which factors determine to have more CNSs or more genes?

The frequency of CNSs in the UTRs was observed to be higher than the genomic coverage for the UTRs in the reference genomes, thus shows a stronger selective pressure on the CNSs located in the UTRs. Most of the UTR CNSs were found in 3′-UTRs for the grass-specific CNSs .This finding is consistent with earlier reports of conservation in the 3′-UTRs (Duret et al. 1993; Lipman 1997; Grzybowska et al. 2001, Siepel et al. 2005). In addition, the enriched conservation in UTR was observed for genes in DNA binding proteins (Duret et al. 1993). However, it is likely that 3′-UTR conservation found in this study could be involved in posttranscriptional regulatory mechanisms as well directing subcellular localization, transcript stability or translatability. In accordance with this assumption, we observed a lower nucleosome occupancy probability for the 3′-UTR CNSs compared with CNSs in other regions of the genome.

The drop in A+T content near the borders of the CNSs is a feature that is also seen in animals (Walter et al. 2005; Vavouri et al. 2007). Therefore, it is possible that this orientation of nucleotides such as the drop of the A+T content near the boundaries is an important feature for the CNS function. This shows a required functional property of CNSs, even though the reason for this CNS layout conservation between animals and plants is not yet known. But one candidate explanation lies with A+T content and the nucleosome formation.

With the nucleosome positioning analysis it was observed that the CNSs tested, showed high nucleosome occupancy probability in and around the CNSs implying that CNSs may have a higher probability to form nucleosomes. The finding by Bai and Morozov (2010) and Jiang and Pugh (2009) stating that nucleosome positioning is related to gene regulation gives evidence to support the fact that CNSs may be involved in transcriptional regulation of their target genes. Also Tirosh and Barkai (2008) reported that high nucleosome occupancy near transcription start site is associated with transcription and that regulatory elements with high occupancy are more responsive to external and internal signals in the yeast genome. These findings further support the view of CNSs playing a regulatory role. One important feature in the result is the A+T increased flanking regions just before the drop of A+T content. These A+T increased regions level off to the genomic average of the reference genome in the study. It can be argued that the regions with high A+T content do not fold into nucleosomes, rather they can be acting as linker regions with low G+C content (Nishida 2012) that is adjacent to nucleosomes. We also found that the A+T drop may not be related to recombination rate variation in the genome.

Gene enrichment analysis carried out for grass and monocot-specific CNSs suggests that CNSs tend to locate close to genes involved in DNA binding, transcription regulation, and transcription factor activity. Animal genome analyses demonstrated that CNSs are found near genes involved in regulation of transcription and development (Sandelin et al. 2004; Shin et al. 2005; Venkatesh et al. 2006; Matsunami and Saitou 2012; Babarinde and Saitou 2013). This finding for the lineage-specific CNSs also agrees with animal CNS studies reported so far. One interesting feature to note down is that lineage-specific genes and lineage-specific CNSs have different functional classifications. Lineage-specific genes were found to be plant defense related, whereas lineage-specific CNSs are related to regulation of transcription and development. Therefore, it appears that lineage CNSs and genes are functioning in two diverse arenas to ensure thorough overall accurate functioning of the plant. Interestingly, Babarinde and Saitou (2013) reported that two underrepresented terms for their GO analysis for CNSs include categories related to stimulus and defense.

Even though we considered the closest gene to the CNSs as the likely target gene, it is noteworthy that without experimental support and evidence it is hard to establish the actual target genes of CNSs. Also it is important to note that there are exceptions to the above-mentioned scenario. As reported by Lettice et al. (2003), a regulator designated as ZRS responsible for early spatiotemporal expression pattern in the limb of tetrapods lies in intron 5 of *Lmbr1* gene where the target gene *Shh* lies 1 Mb away from the enhancer.

The result achieved for grass and monocot CNSs and also for certain grouping for eudicots and angiosperms are consistent with the established phylogeny of plants (Angiosperm Phylogeny Group 2003; Bennetzen et al. 2012) thus agrees with the expectation of CNSs being orthologous in different species. This analysis also shows that CNSs can be used to construct species trees provided that concatenated sequence lengths are of considerable lengths with enough informative sites.

In this study, we identified 27 eudicot, 204 monocot, 6,536 grass, 19 angiosperm, and 2 vascular plant lineage-specific CNSs that originated in their respective common ancestors. We also observed a stronger constraint on CNSs located on UTRs. The CNSs are flanked by genes involved in transcription regulation and also a drop of A+T was observed near the borders of the CNSs. Further, the CNSs showed a high nucleosome occupancy probability. This study provides candidates of regulatory elements that can be experimentally tested for their potential functionality. These findings along with other investigations on plant CNSs will help to establish an understanding to shape the regulatory landscape of plants, governed by CNSs.

## Supplementary Material

Supplementary figures S1–S8 and tables S1–S5 are available at *Genome Biology and Evolution* online (http://www.gbe.oxfordjournals.org/).

Supplementary Data
