# Supplementary material for: Lineage-Specific Conserved Noncoding Sequences of Plant Genomes: Their Possible Role in Nucleosome Positioning
Source: Genome Biol Evol. 2014 Sep 5;6(9):2527–42. doi: 10.1093/gbe/evu188 (PMC4202324; doi:10.1093/gbe/evu188)
Supplement: Supplementary Data [file supp_6_9_2527__index.html]

Lineage specific conserved noncoding sequences of plant genomes: their possible role in nucleosome positioning — Lineage-Specific Conserved Noncoding Sequences of Plant Genomes: Their Possible Role in Nucleosome Positioning — Supplementary Data 

# Lineage-Specific Conserved Noncoding Sequences of Plant Genomes: Their Possible Role in Nucleosome Positioning

## Supplementary Data

files

**Files in this Data Supplement:**

- Supplementary Data - pdf file
- Supplementary Data - txt file
- Supplementary Data - txt file
- Supplementary Data - txt file
