# Supplementary material for: Lineage-Specific Conserved Noncoding Sequences of Plant Genomes: Their Possible Role in Nucleosome Positioning
Source: Genome Biol Evol. 2014 Sep 5;6(9):2527–42. doi: 10.1093/gbe/evu188 (PMC4202324; doi:10.1093/gbe/evu188)
Supplement: Supplementary Data [file supp_evu188_Hettiarachchi_Suppl_Figs_Tabs.pdf]

Supplementary figure 1: Length and conservation level relationship for lineage specific CNSs. (A) Length and conservation level for grass specific CNSs. (B) Length and conservation level for monocot specific CNSs. (C) Length and conservation level for dicot specific CNSs. (D) Length and conservation level for angiosperm specific CNSs.

(A)

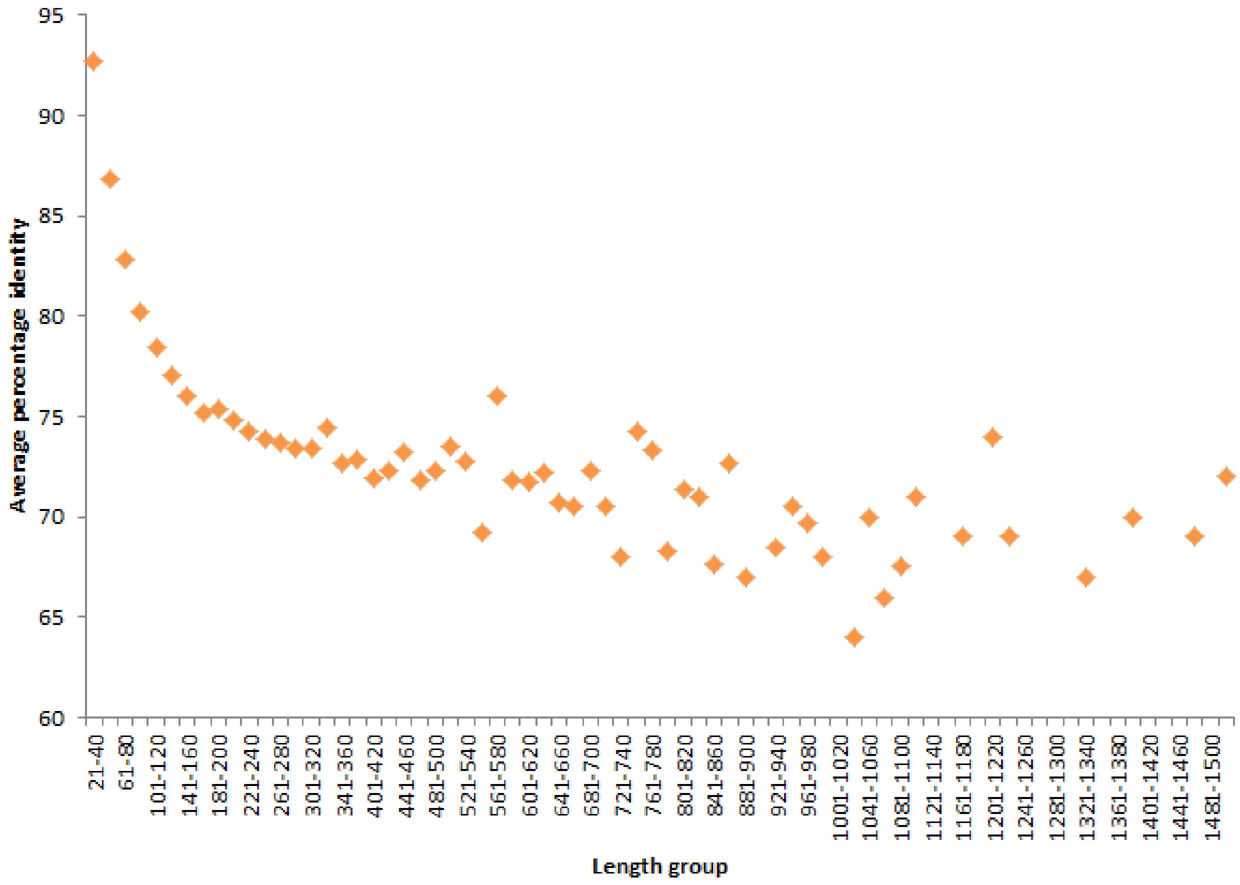

(B)

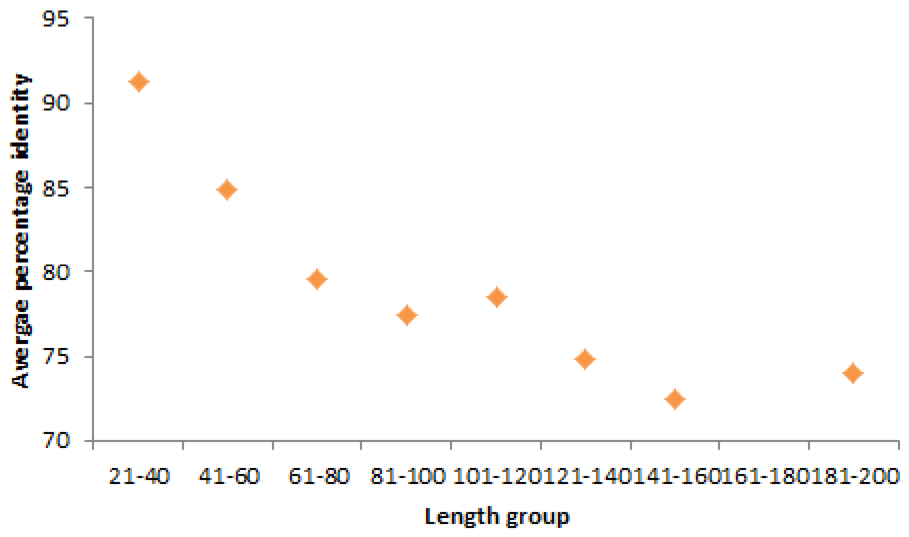
95
90
85
80
75
70
Avergae percentage identity
21-40
41-60
61-80
81-100
101-120
121-140
141-160
161-180
181-200
Length group

(C)

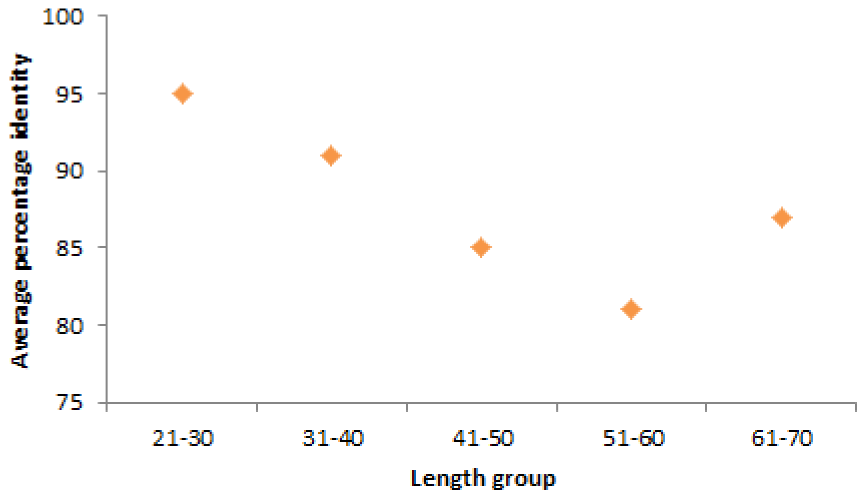
100
95
90
85
80
75
Average percentage identity
21-30
31-40
41-50
51-60
61-70
Length group

(D)

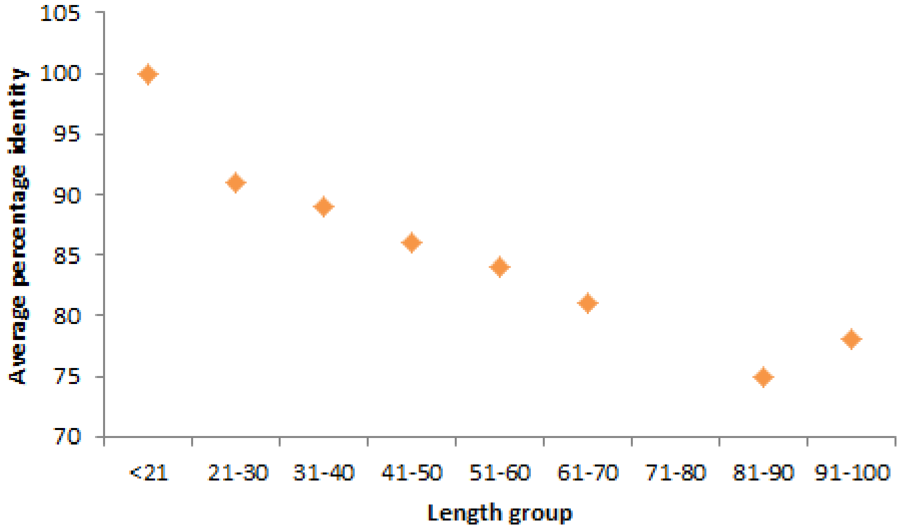
105
100
95
90
85
80
75
70
Average percentage identity
<21
21-30
31-40
41-50
51-60
61-70
71-80
81-90
91-100
Length group

Supplementary figure 2: Examples of multiple sequence alignments (A) Angiosperm specific CNSs. (B) Dicot specific CNSs. (C) Monocot specific CNSs. (D) Grass specific CNSs.

(A)

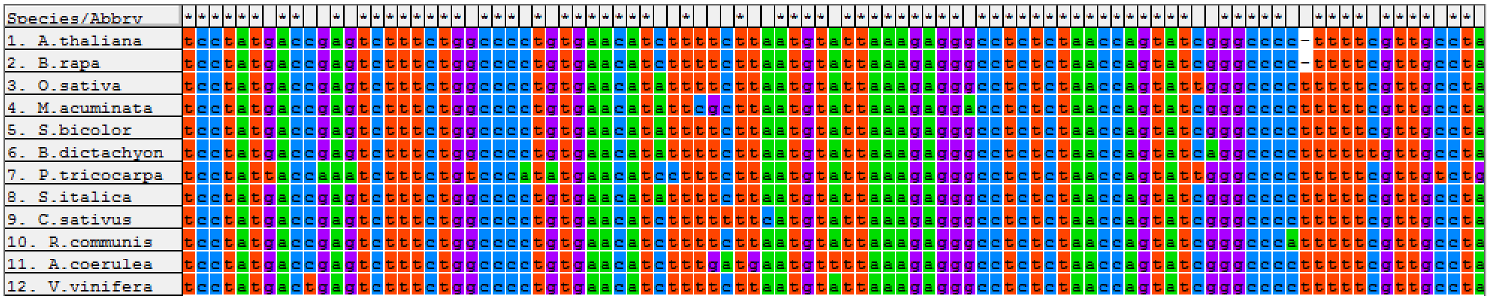

(B)

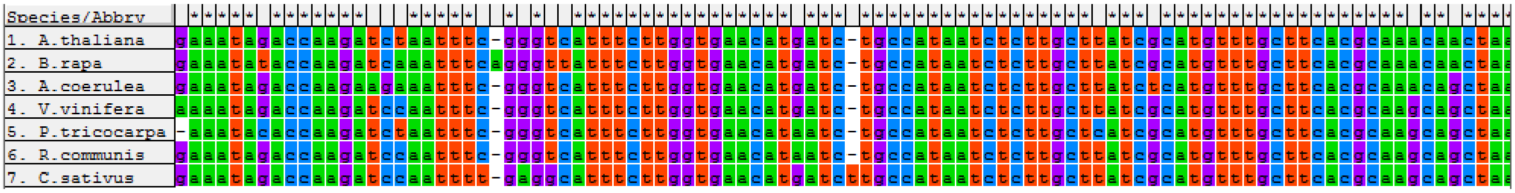

(C)

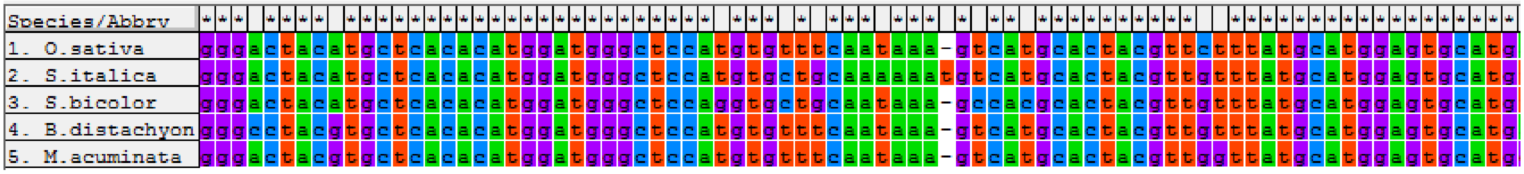

(D)

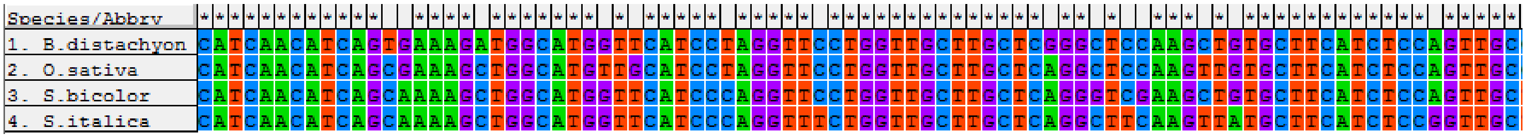

Supplementary figure 3: Distribution of grass specific CNSs on *O.sativa japonica* chromosomes. Y axis – genomic location of the CNSs (Mb), X axis – cumulative frequency of the CNSs. The clearly visible clusters of CNSs are encircled.

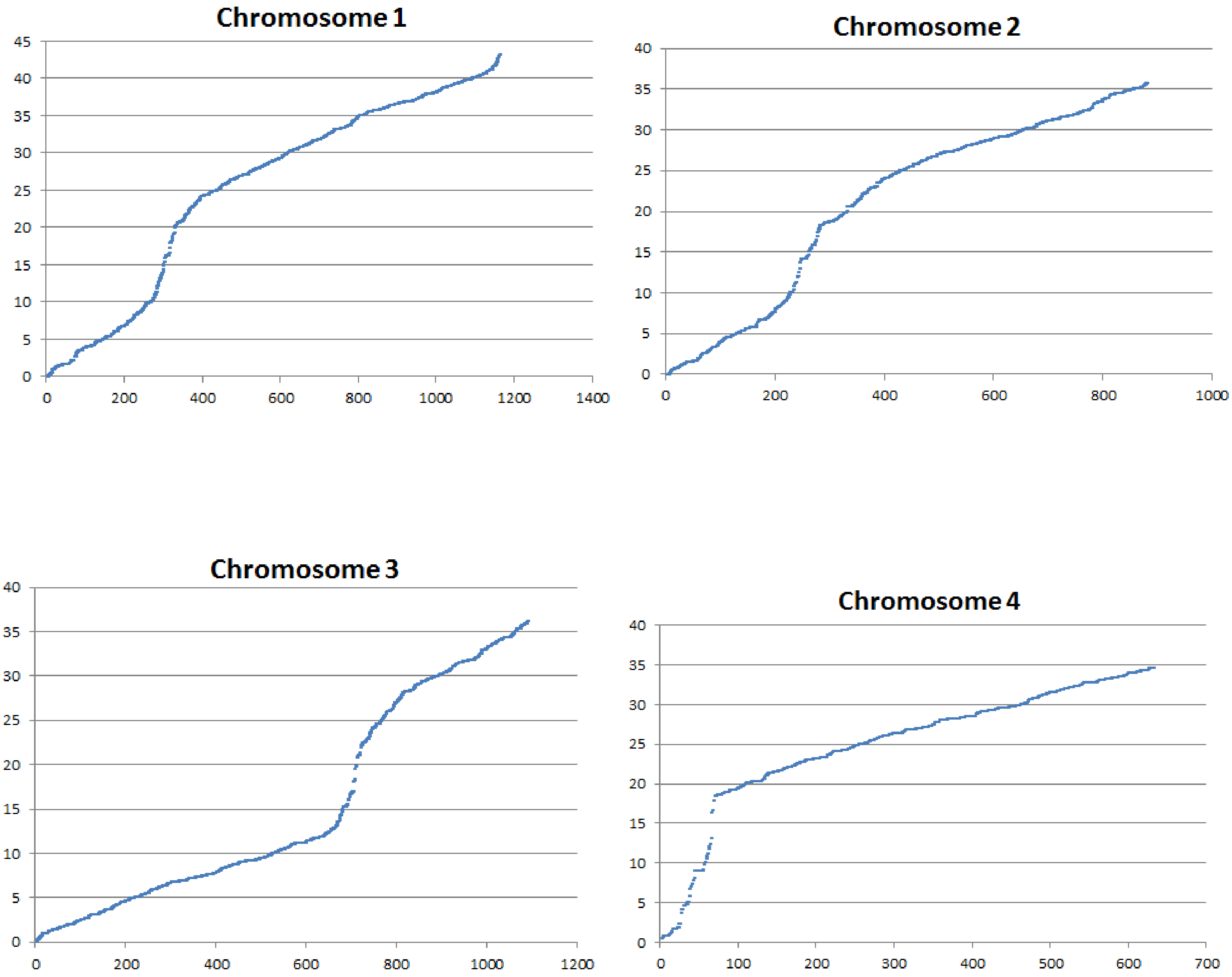

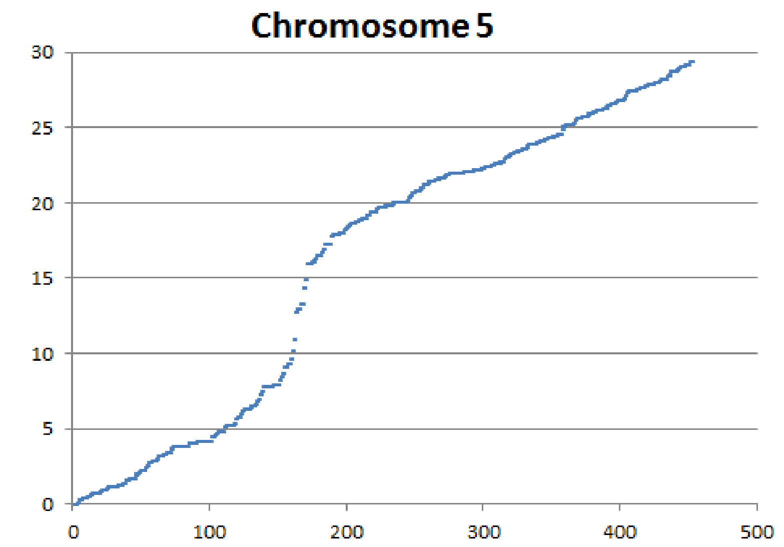
Chromosome 5
0 5 10 15 20 25 30
0 100 200 300 400 500

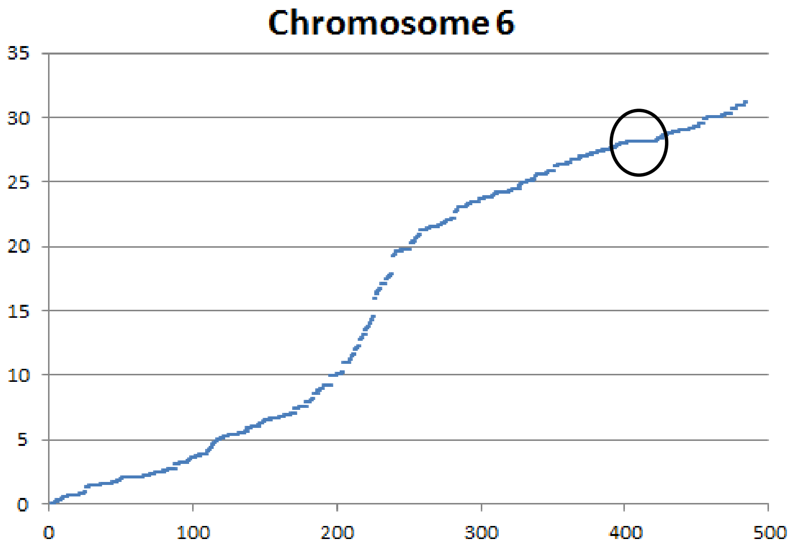
Chromosome 6
0 5 10 15 20 25 30 35
0 100 200 300 400 500

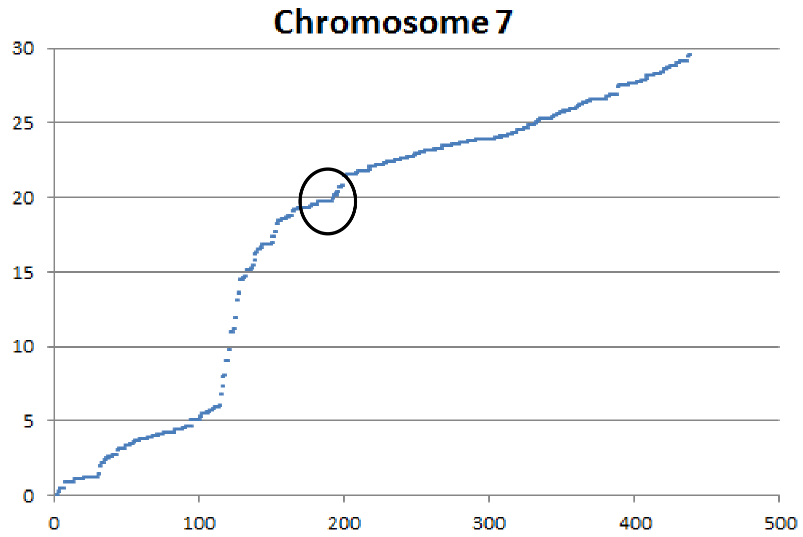
Chromosome 7
0 5 10 15 20 25 30
0 100 200 300 400 500

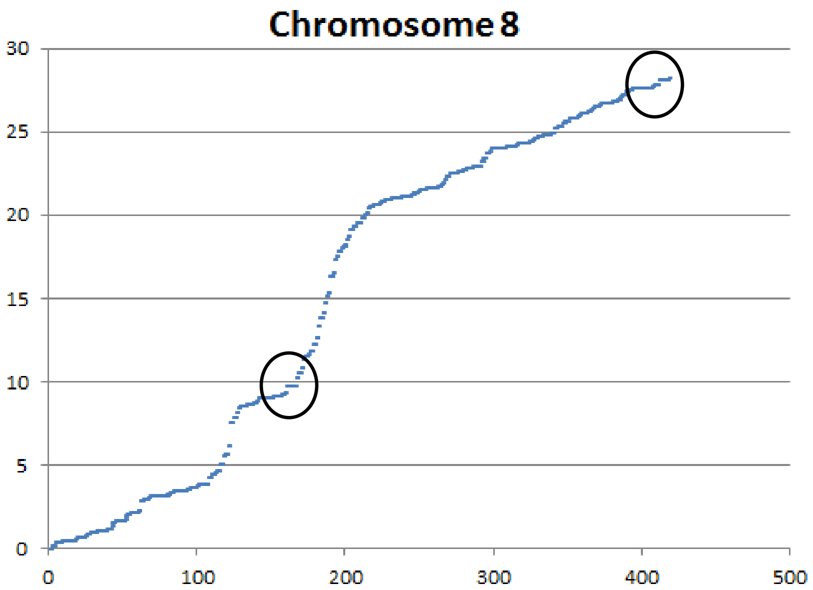
Chromosome 8
0 5 10 15 20 25 30
0 100 200 300 400 500

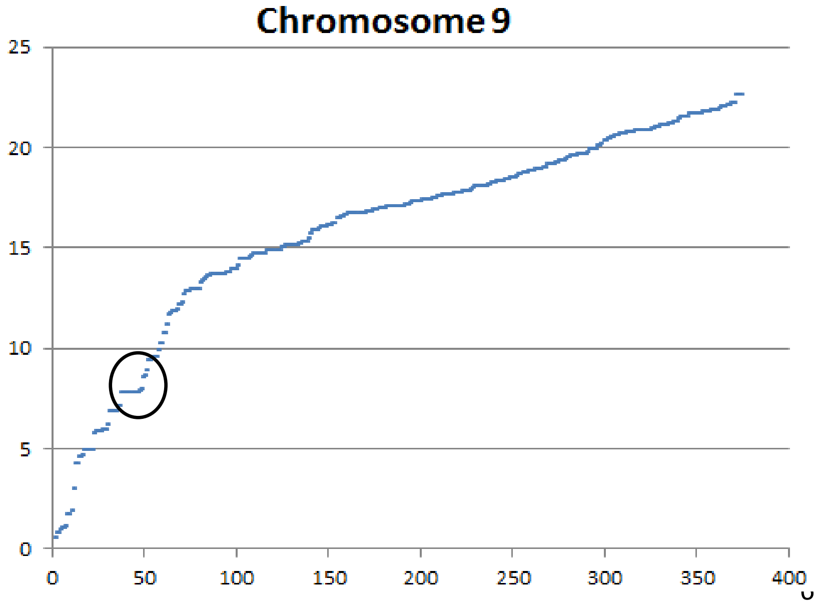
Chromosome 9
0 5 10 15 20 25
0 50 100 150 200 250 300 350 400

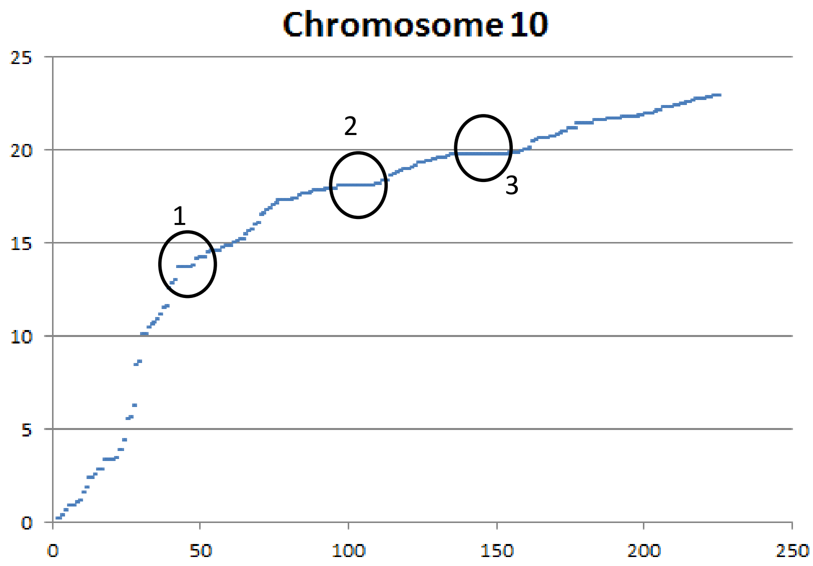
Chromosome 10
0 5 10 15 20 25
0 50 100 150 200 250
1
2
3

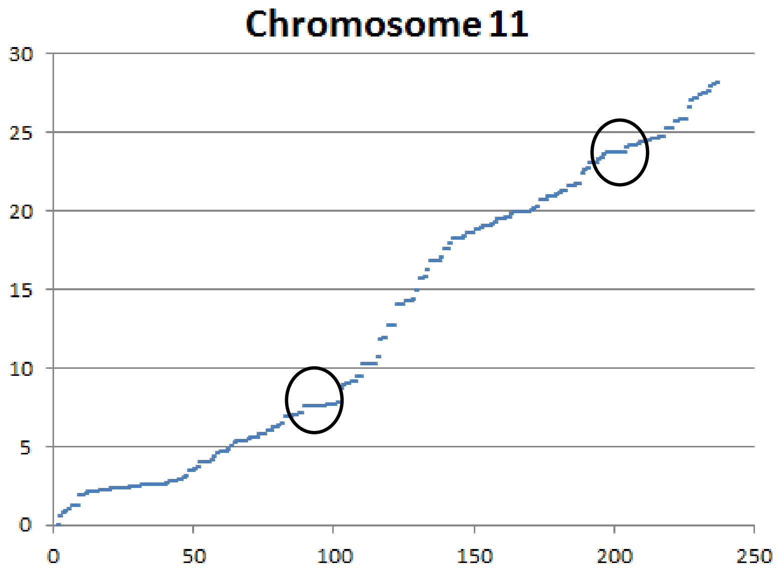
Chromosome 11
30
25
20
15
10
5
0
0
50
100
150
200
250

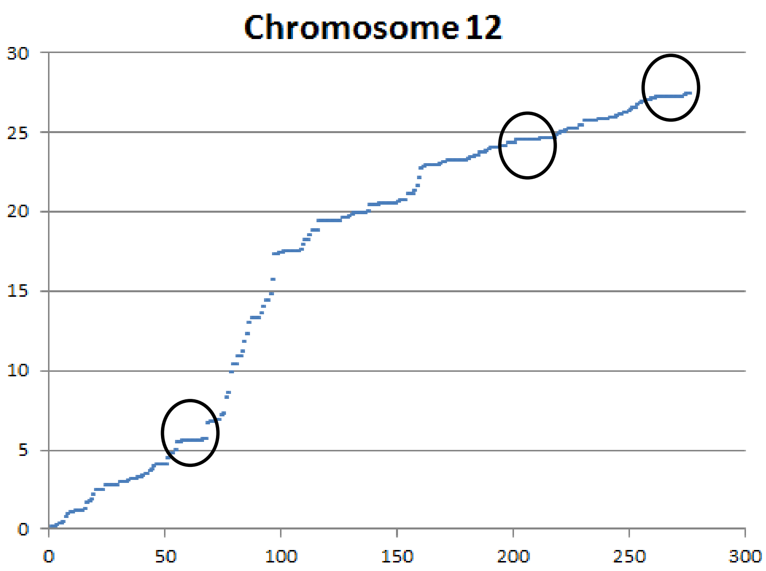
Chromosome 12
30
25
20
15
10
5
0
0
50
100
150
200
250
300

Supplementary figure 4: Distribution of monocot specific CNSs on *O.sativa japonica* chromosomes. Y axis – genomic location of the CNSs (Mb), X axis – cumulative frequency of the CNSs.

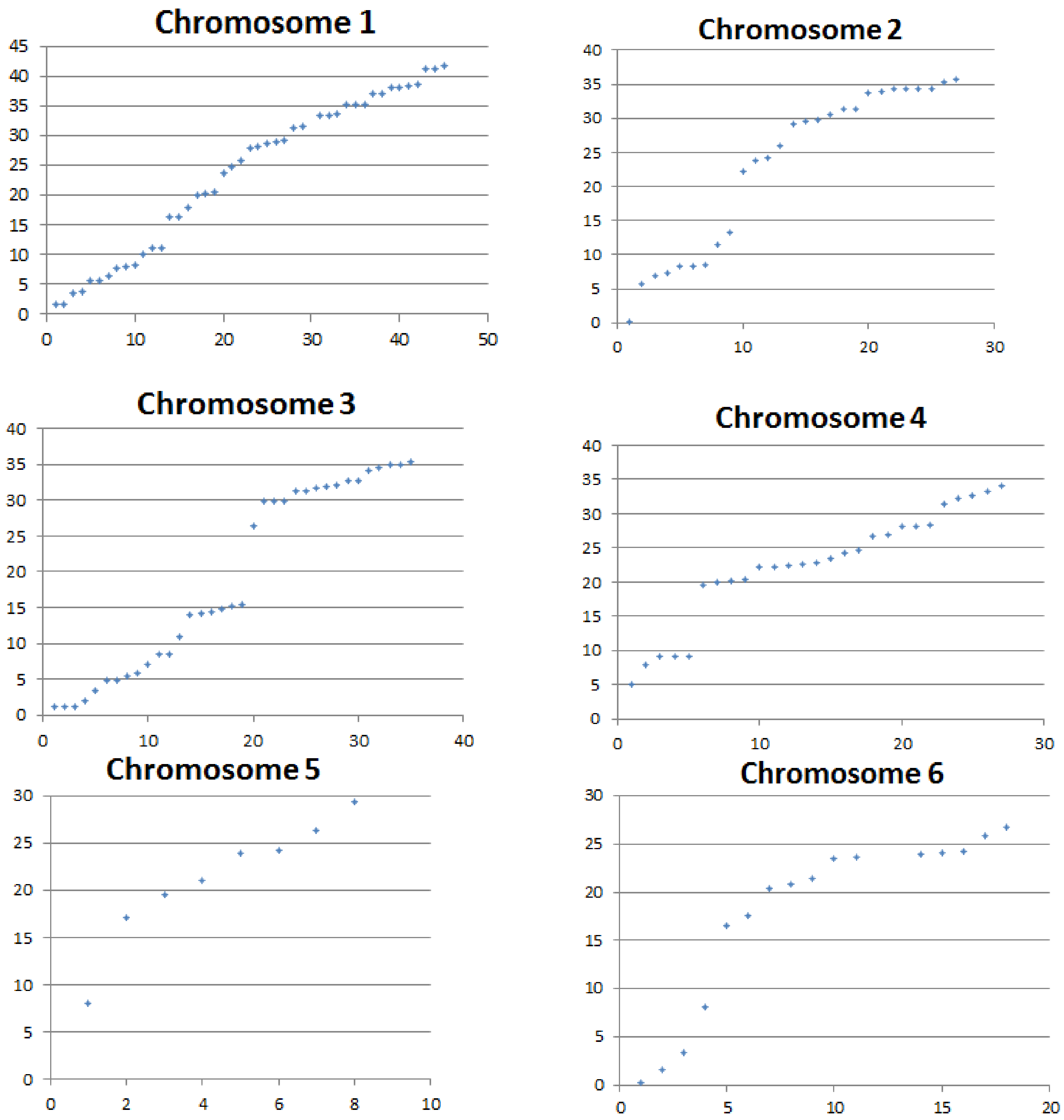

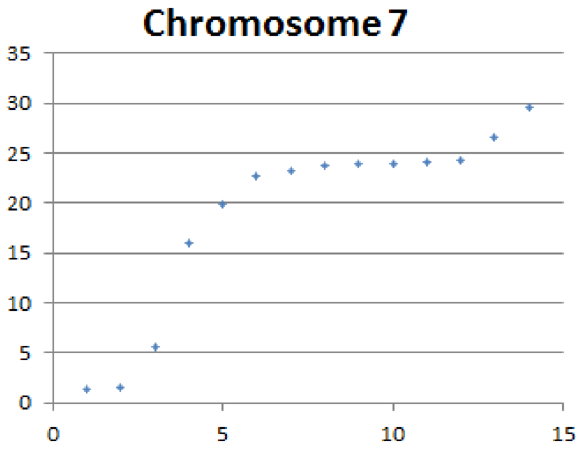
Chromosome 7
35
30
25
20
15
10
5
0
0
5
10
15

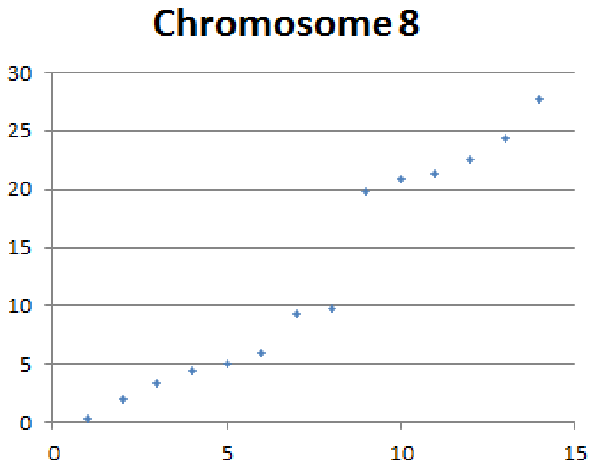
Chromosome 8
30
25
20
15
10
5
0
0
5
10
15

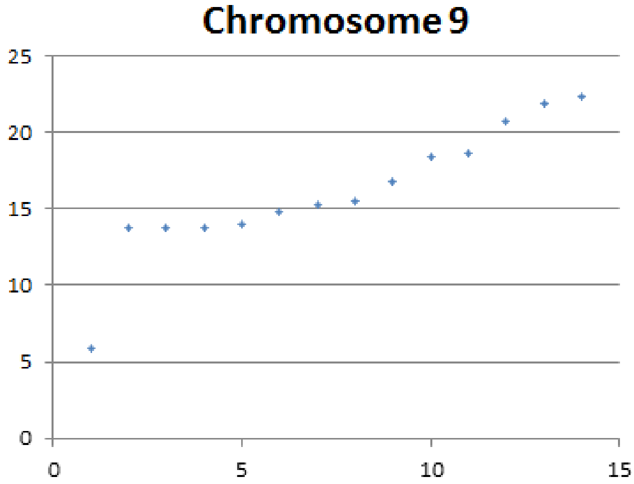
Chromosome 9
25
20
15
10
5
0
0
5
10
15

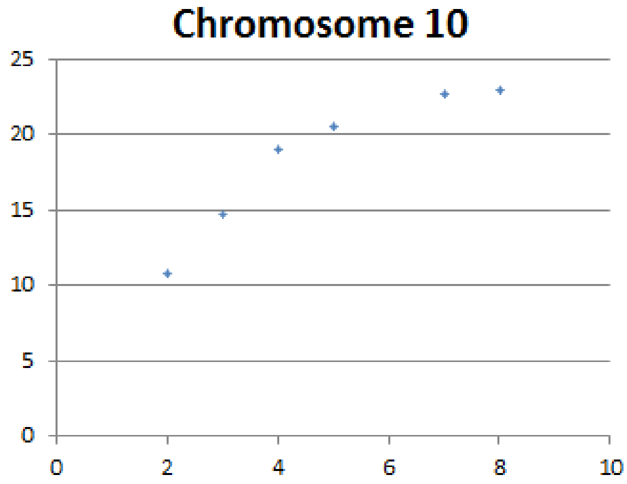
Chromosome 10
25
20
15
10
5
0
0
2
4
6
8
10

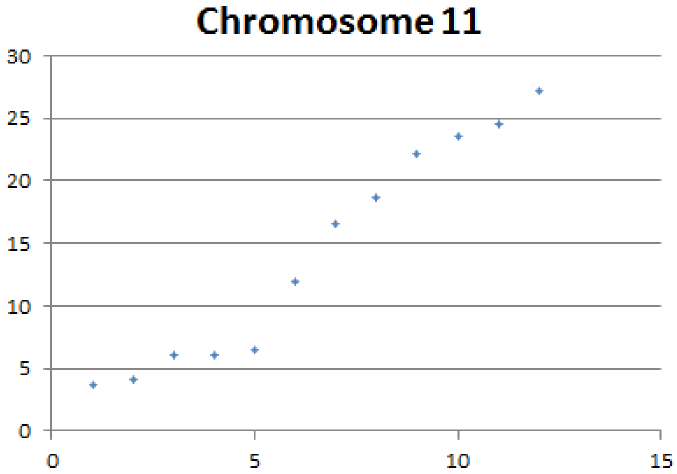
Chromosome 11
30
25
20
15
10
5
0
0
5
10
15

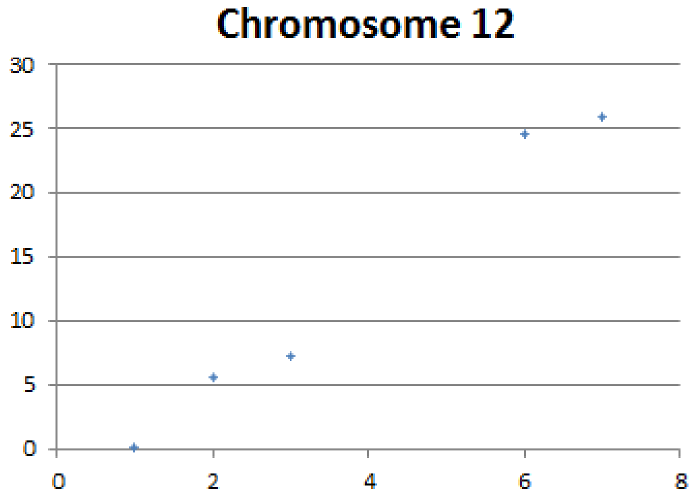
Chromosome 12
30
25
20
15
10
5
0
0
2
4
6
8

Supplementary figure 5: (A) Distribution of A+T content in the flanking regions and within monocot specific CNSs, black line – average A+T content in rice genome. (B) Nucleosome occupancy probability for monocot specific CNSs including flanking regions. 0th nucleotide position represents the center of each CNS and also that of the random samples. Blue, Red and green graphs respectively show nucleosome occupancy probabilities of the CNSs, random sample with same AT content as CNSs and the random sample without specific AT preference.

(A)

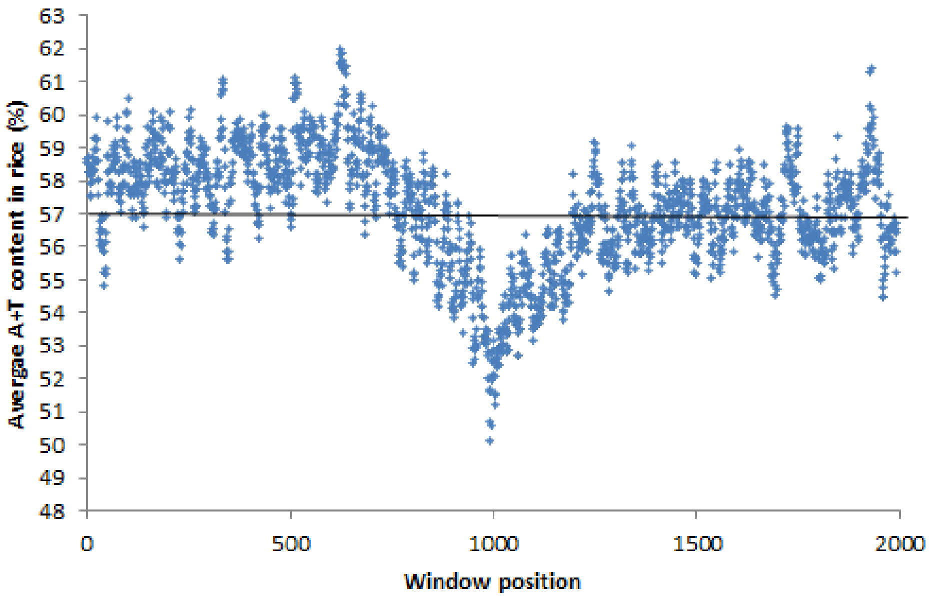

(B)

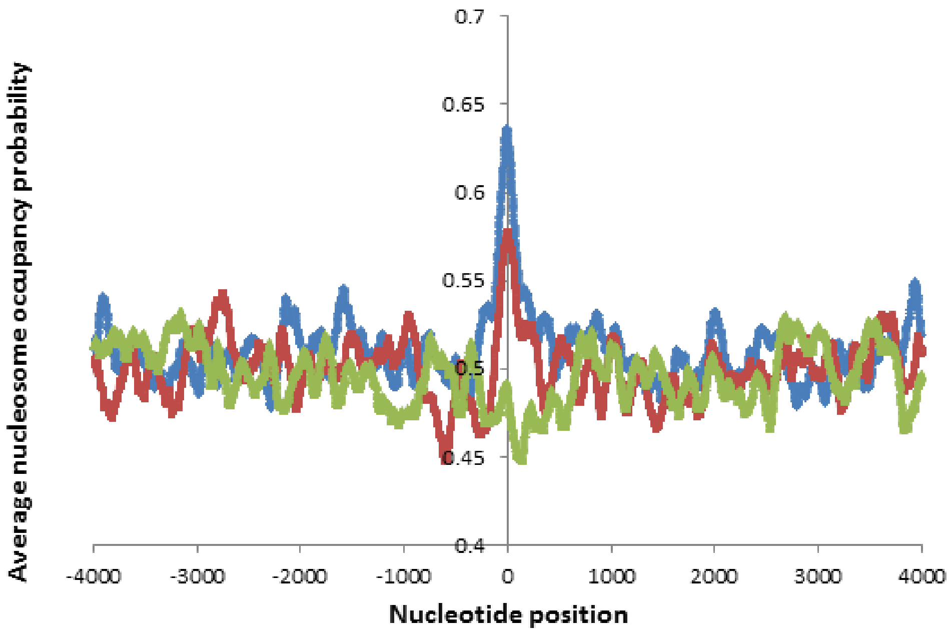

Supplementary figure 6: The phylogenetic trees of the CNSs. The concatenated lineage specific CNSs were used to construct the phylogenetic trees with neighbor-joining method. (A) Phylogenetic tree constructed for grasses with grass specific CNSs (B) Phylogenetic tree constructed for all monocots in the study with monocot specific CNSs. (C) Phylogenetic tree constructed for dicots in the study with dicot specific CNSs. (D) Phylogenetic tree constructed for all angiosperms in the study with angiosperm specific CNSs.

(A)

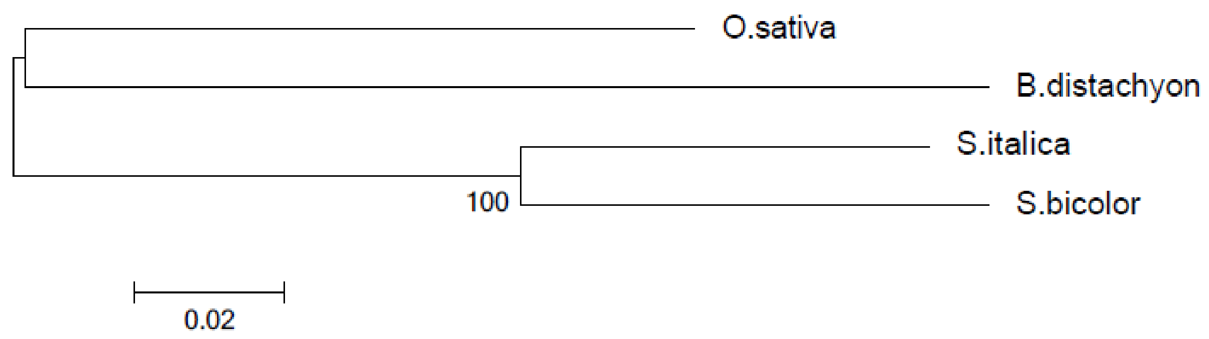

(B)

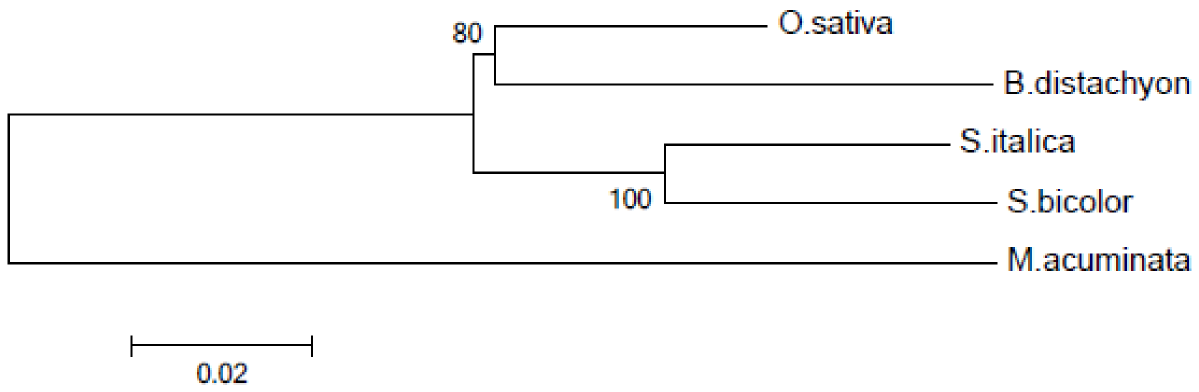

(C)

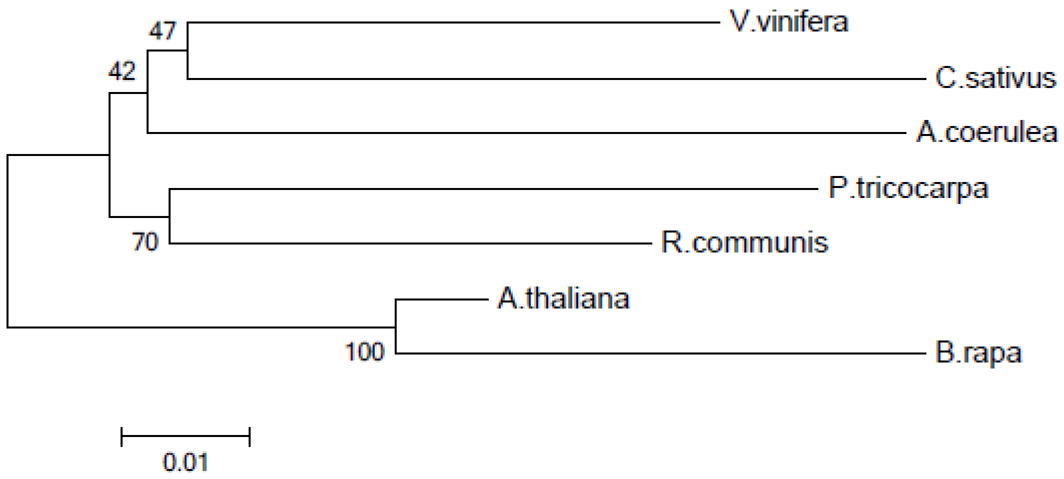
47
42
70
100
V.vinifera
C.sativus
A.coerulea
P.tricocarpa
R.communis
A.thaliana
B.rapa
0.01

(D)

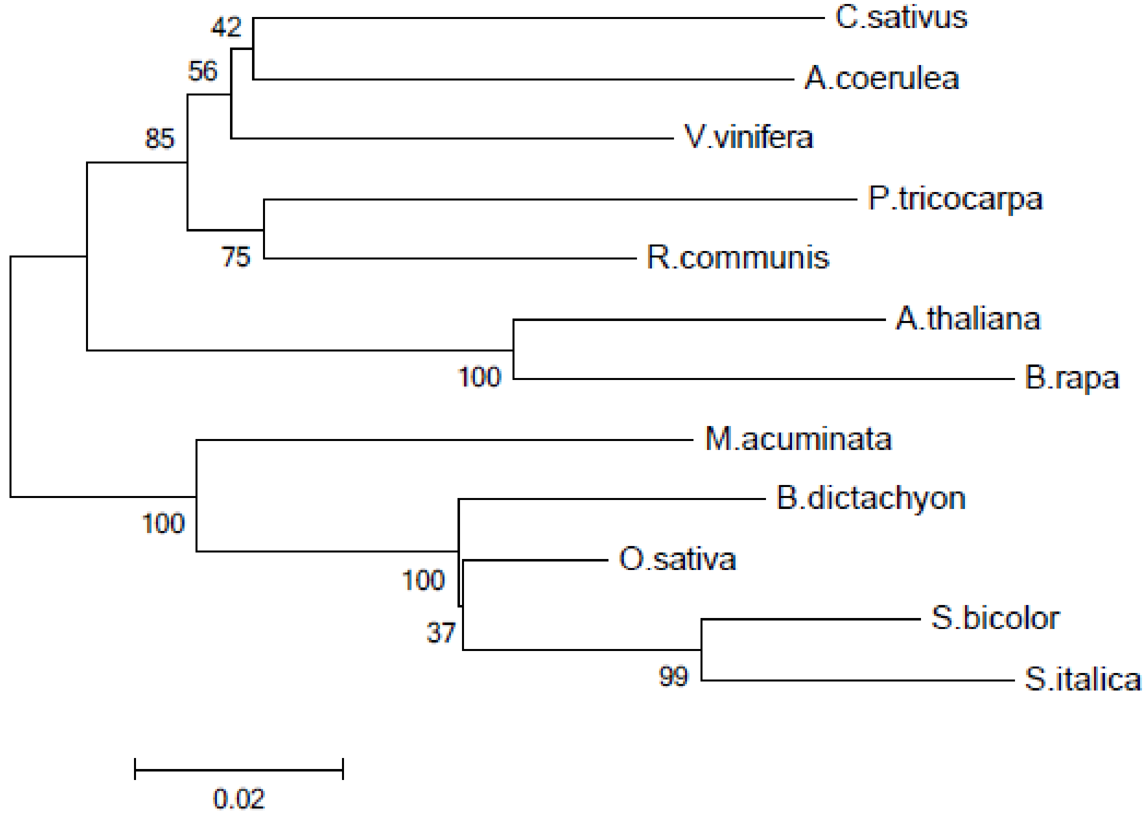
42
56
85
75
100
100
100
37
99
C.sativus
A.coerulea
V.vinifera
P.tricocarpa
R.communis
A.thaliana
B.rapa
M.acuminata
B.dictachyon
O.sativa
S.bicolor
S.italica
0.02

Supplementary figure 7: The phylogenetic tree for lineage common CNSs.

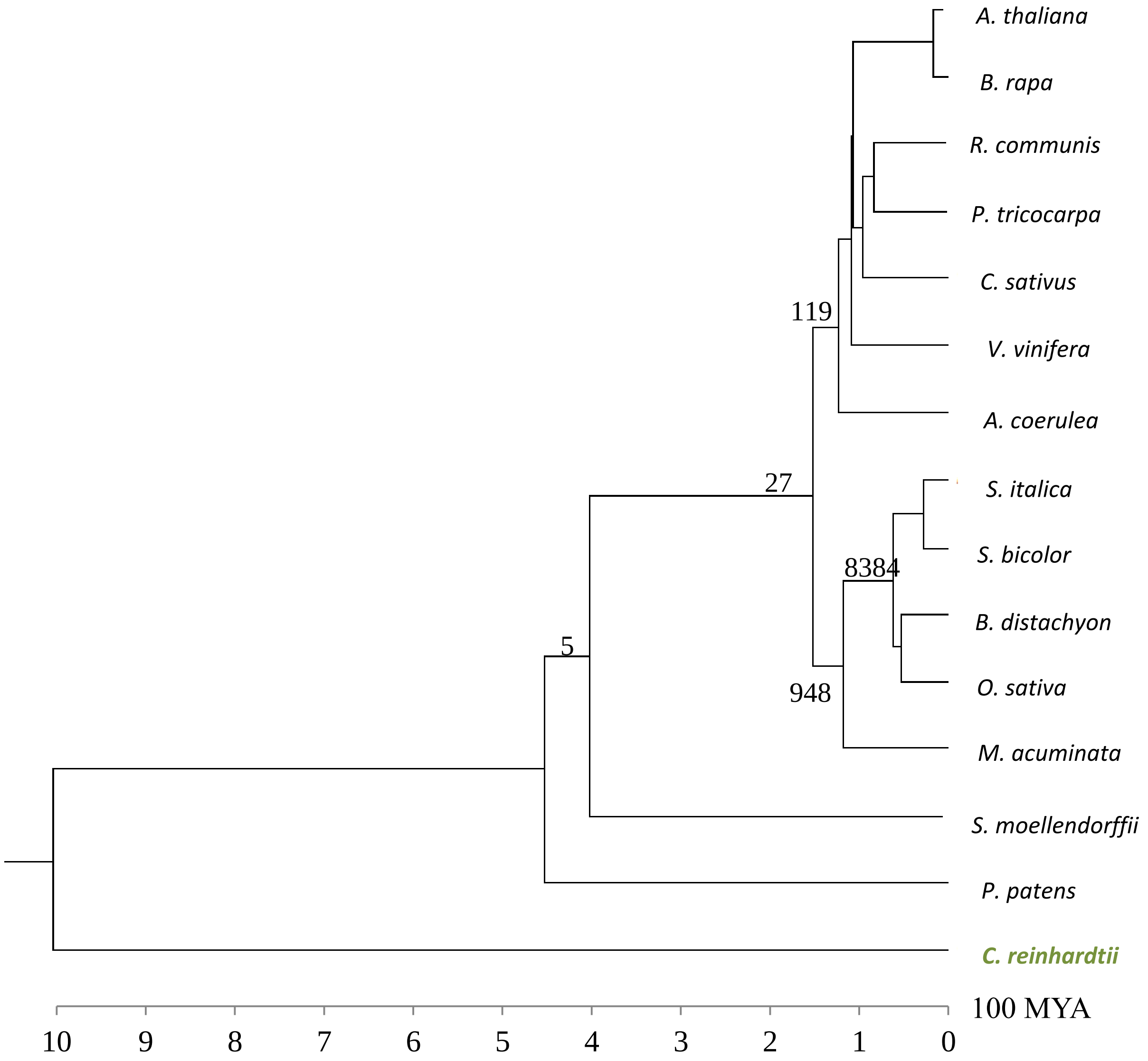

Supplementary figure 8: Nucleosome occupancy probability for grass specific UTR, Intergenic and Intronic CNSs. 0th nucleotide position represent the center of the CNSs. (A) Nucleosome occupancy probability for grass specific UTR CNSs. Purple, green and red graphs respectively show nucleosome occupancy probabilities of the 5' UTR CNSs, 3' UTR CNSs and random samples with same AT content as CNSs. (B) Nucleosome occupancy probability for grass specific intergenic CNSs. Blue and red graphs show the nucleosome probabilities for intergenic CNSs and random samples. (C) Nucleosome occupancy probability for grass specific intronic CNSs. Blue and red graphs show the nucleosome probabilities for intronic CNSs and random samples.

(A)

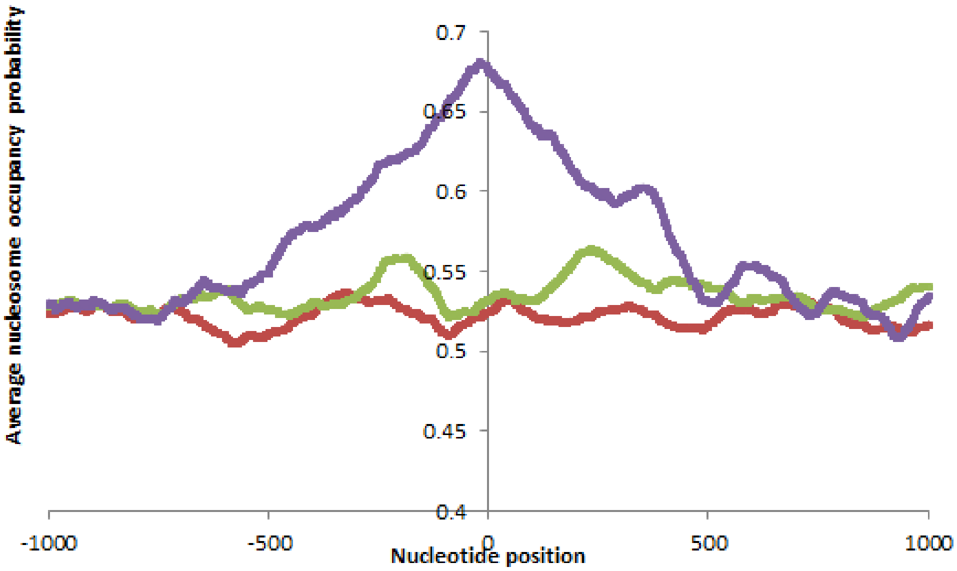

(B)

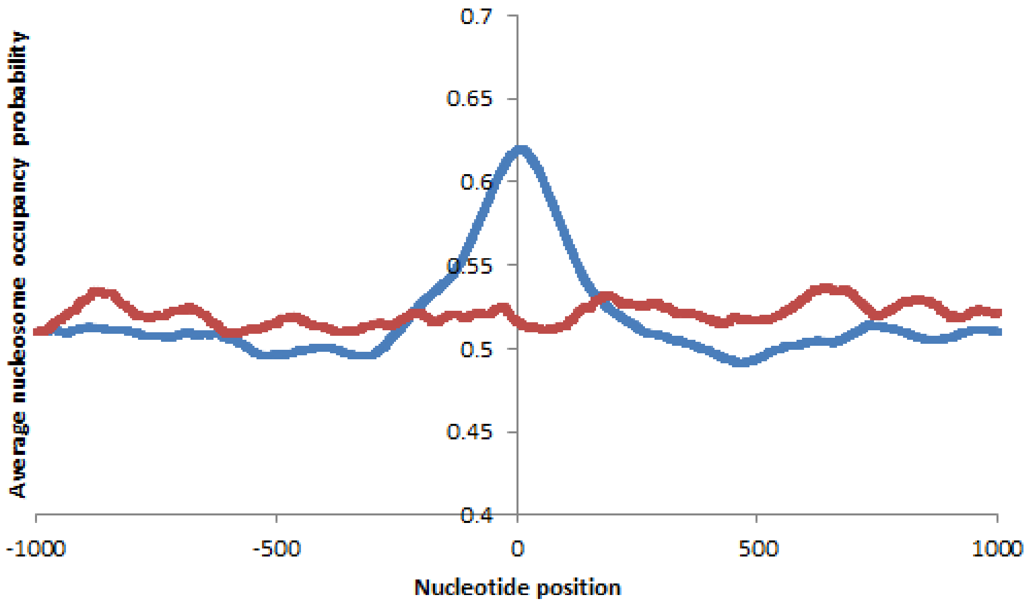
0.7
0.65
0.6
0.55
0.5
0.45
0.4
-1000
-500
0
500
1000
Nucleotide position
Average nucleosome occupancy probability

(C)

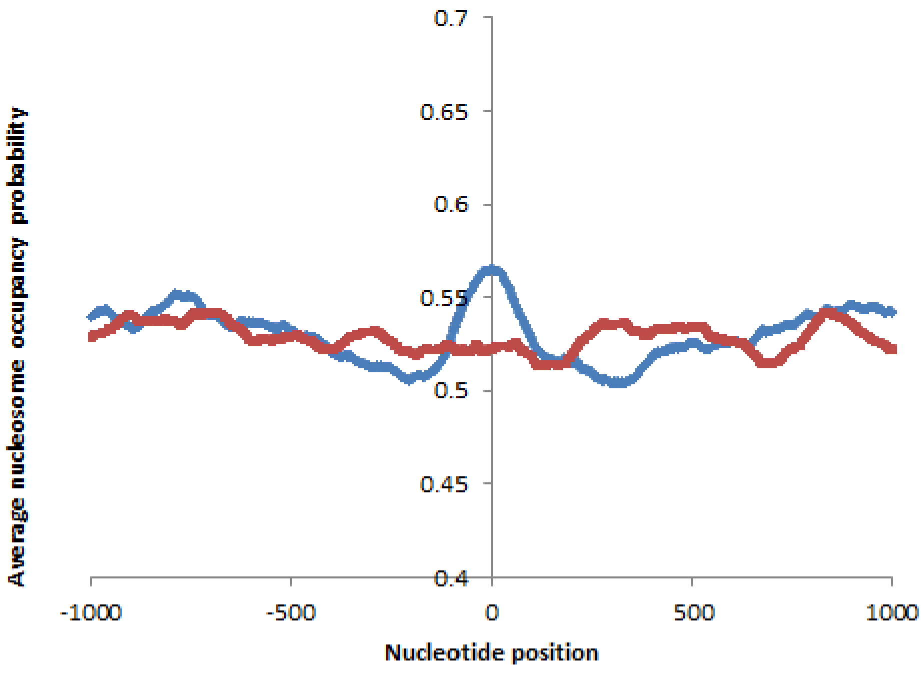
0.7
0.65
0.6
0.55
0.5
0.45
0.4
-1000
-500
0
500
1000
Nucleotide position
Average nucleosome occupancy probability

Supplementary Table 1: Numbers on ancestral CNSs found between *C. reinhardtii* and other species.

Query	Subject	Number of ancestral CNSs
C. reinhardtii	*A. thaliana*	210
	B. rapa	368
	R. communis	649
	P. tricocarpa	677
	C. sativus	233
	V. vinifera	156
	A. coerulea	198
	S. italica	259
	S. bicolor	911
	B. distachyon	482
	O. sativa japonica	708
	M. acuminate	1049
	S. moellendorffii	1741
	P. patens	958

Supplementary Table 2: Gene enrichment analysis for 6536 random genes of *O. sativa japonica*

Functional group	Percentage of genes in the group	P-value
Root hair cell differentiation	100.0	1.7E-8
Tricoblast maturation	100.0	1.7E-8
Cell maturation	100.0	1.7E-8
Trichoblast differentiation	100.0	2.2E-8
Root epidermal cell differentiation	100.0	3.3E-8
Developmental maturation	100.0	3.8E-8
Epidermal cell differentiation	100.0	5.1E-7
Cell growth	75.0	6.9E-4
Regulation of cell size	75.0	7.7E-4
Growth	75.0	9.1E-4
Cell tip growth	50.0	1.5E-2
Leaf morphogenesis	50.0	2.3E-2
Shoot morphogenesis	50.0	3.2E-2
Leaf development	50.0	3.9E-2
Glycoprotein	50.0	4.0E-2
Phyllome development	50.0	4.3E-2
Shoot development	50.0	5.9E-2
Post embryonic development	25.0	1.0E-0
GPI-anchor	25.0	1.0E-0
Endomembrane system	25.0	1.0E-0

Supplementary Table 3: (A) Under-represented GO terms related to catabolic processes. (B) Under-represented GO terms related to enzymatic and structural processes.

(A)

Functional group	Percentage of genes in the group	P-value
Modification dependent protein catabolic process	98.5	2.8E-92
Macromolecule catabolic process	98.5	2.8E-92
Cellular protein catabolic process	98.5	5.1E-92
Ubl conjugation pathway	81.8	6.5E-90
Proteolysis	98.5	6.8E-74
Ubiquitin-protein ligase activity	59.1	3.5E-53
Amino-acid ligase activity	59.1	2.3E-51
Ubiquitin-ligase-complex	37.9	9.2E-23
U box domain	25.8	1.9E-27
Protein ubiquitination	28.8	3.0E-22

(B)

Functional group	Percentage of genes in the group	P-value
Glycoside hydrolase	65.7	9.8E-40
Glycosidase	60.0	2.3E-33
Signaling pathway	57.1	9.0E-18
Glycoprotein	48.6	6.2E-16
Related to extra cellular region	48.6	6.5E-9
Cation binding	65.7	1.1E-7
Cell wall	31.4	1.4E-6
External encapsulating structure	31.4	1.6E-6
Apoplast	22.9	3.4E-6
Cell wall biogenesis/degradation	17.1	8.5E-6

Supplementary Table 4: Motif overrepresentation data for lineage specific CNSs. This table gives the motifs with highest statistical significance. Motifs identified by MEME for dicot specific CNSs have a higher E-value.

	Motif logo	E-value	GO term
Grass specific	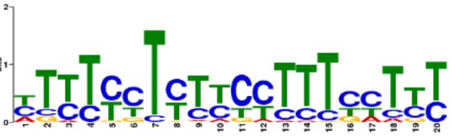	4.2e-024	TF activity
	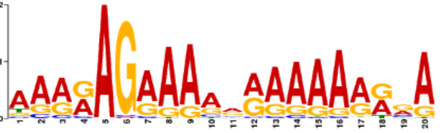	4.5e-023	TF activity
	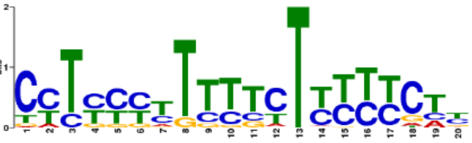	2.3e-023	TF activity
	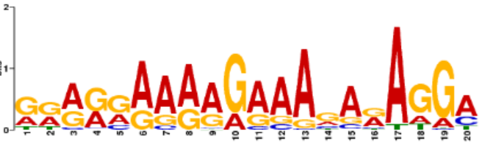	6.2e-013	DNA binding
	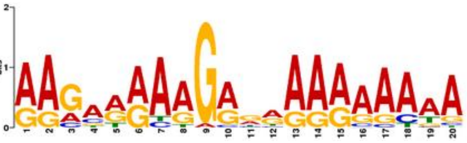	3.3e-015	TF activity
Monocot specific	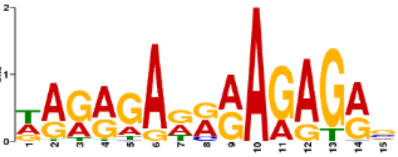	8.5e-023	TF activity
	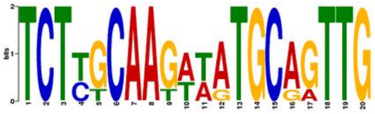	1.5e-001	GO term unknown
	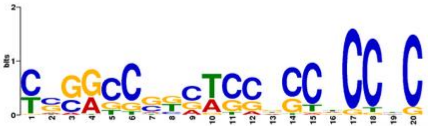	2.8e-001	DNA binding
Dicot specific	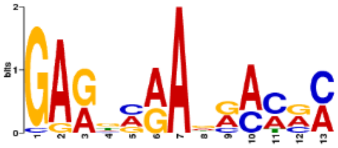	1.8e+002	GO unknown

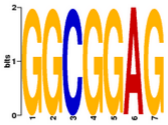	7.8e+003	GO unknown
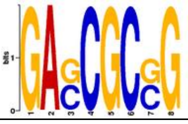	2.4e+004	GO unkonwn

Supplementary Table 5: Searches performed for all pairs of species in the study. The searches were performed with the same cut off e-value for lineage specific CNSs. The best hits were selected and concatenated when determining CNSs common for each lineage in the common ancestor.

Search	Number of CNSs
Arabidopsis-Brassica	57132
Ricinus-Populus	30355
Cucumis-Ricinus	4983
Cucumis-Populus	4007
Vitis-Cucumis	2581
Vitis-Populus	7109
Vitis-Ricinus	5479
Vitis-Brassica	2534
Vitis-Arabidopsis	945
Aquilegia-Vitis	3241
Aquilegia-Cucumis	1603
Aquilegia-Populus	2488
Aquilegia-Ricinus	2729
Aquilegia-Brassica	2169
Aquilegia-Arabidopsis	834
Arabidopsis-Ricinus	1370
Arabidopsis-Populus	1410
Arabidopsis-Cucumis	778
Brassica-Ricinus	9981
Brassica-Populus	8150
Brassica-Cucumis	3598
Cucumis-Ricinus	4983
Cucumis-Populus	4007
Setaria-Sorghum	30134
Oryza-Brachypodium	20323
Setaria-Brachypodium	17386
Setaria-Oryza	21717
Sorghum-Brachypodium	14723
Sorghum-Oryza	18029
Musa-Brachypodium	1693
Musa-Oryza	2278
Musa-Sorghum	830
Musa-Setaria	2132
Musa-Brassica	4045
Musa-Ricinus	6321
Musa-Populus	3345

Musa-Cucumis	1474
Musa-Vitis	3177
Musa-Aquilegia	496
Musa-Arabidopsis	1412
Oryza-Arabidopsis	608
Oryza-Brassica	1164
Oryza-Ricinus	1194
Oryza-Populus	1250
Oryza-Cucumis	833
Oryza-Vitis	1133
Oryza-Aquilegia	932
Brachypodium-Arabidopsis	488
Brachypodium-Brassica	960
Brachypodium-Ricinus	767
Brachypodium-Populus	821
Brachypodium-Cucumis	647
Brachypodium-Vitis	807
Brachypodium-Aquilegia	616
Sorghum-Arabidopsis	599
Sorghum-Brassica	1339
Sorghum-Ricinus	1412
Sorghum-Populus	1351
Sorghum-Cucumis	824
Sorghum-Vitis	1019
Sorghum-Aquilegia	866
Setaria-Arabidopsis	595
Setaria-Brassica	1498
Setaria-Ricinus	1252
Setaria-Populus	1004
Setaria-Cucumis	962
Setaria-Vitis	1275
Setaria-Aquilegia	1181
Selaginella-Arabidopsis	651
Selaginella-Brassica	2081
Selaginella-Ricinus	2649
Selaginella-Populus	1780
Selaginella-Cucumis	557
Selaginella-Vitis	670
Selaginella-Aquilegia	595
Selaginella-Setaria	696
Selaginella-Sorghum	909
Selaginella-Brachypodium	591
Selaginella-Oryza	766
Selaginella-Musa	3291
Physcomitrella-Arabidopsis	3322
Physcomitrella-Brassica	3241

Physcomitrella-Ricinus	1256
Physcomitrella-Populus	2345
Physcomitrella-Cucumis	1488
Physcomitrella-Vitis	3021
Physcomitrella-Aquilegia	787
Physcomitrella-Setaria	421
Physcomitrella-Sorghum	2968
Physcomitrella-Brachypodium	739
Physcomitrella-Oryza	1583
Physcomitrella-Musa	3332
Physcomitrella-Selaginella	5584
Chlamydomonas-Arabidopsis	100
Chlamydomonas-Brassica	225
Chlamydomonas-Ricinus	330
Chlamydomonas-Populus	355
Chlamydomonas-Cucumis	119
Chlamydomonas-Vitis	93
Chlamydomonas-Aquilegia	112
Chlamydomonas-Setaria	178
Chlamydomonas-Sorghum	564
Chlamydomonas-Brachypodium	438
Chlamydomonas-Oryza	706
Chlamydomonas-Musa	713
Chlamydomonas-Selaginella	747
Chlamydomonas-Physcomitrella	519
Setaria-Sorghum-Brachypodium-Oryza **(Grasses)**	**54353**
Setaria-Sorghum-Brachypodium-Oryza-Musa **(Monocots)**	**6934**
Arabidopsis-Brassica-Ricinus-Populus-Cucumis	19654
Arabidopsis-Brassica-Ricinus-Populus-Cucumis-Vitis	12823
Arabidopsis-Brassica-Ricinus-Populus-Cucumis-Vitis-Aquilegia **(Eudicots)**	**7766**
Arabidopsis-Brassica-Ricinus-Populus-Cucumis-Vitis-Aquilegia-Setaria-Sorghum-Brachypodium-Oryza-Musa-Selaginella **(Vascular plants)**	**8725**
Arabidopsis-Brassica-Ricinus-Populus-Cucumis-Vitis-Aquilegia-Setaria-Sorghum-Brachypodium-Oryza-Musa-Selaginella-Physcomitrella **(Land plants)**	**10820**
Arabidopsis-Brassica-Ricinus-Populus-Cucumis-Vitis-Aquilegia-Setaria-Sorghum-Brachypodium-Oryza-Musa-Selaginella-Physcomitrella-Chlamydomonas **(plants)**	**3246**
